# Interplay of Hydration and Protonation Dynamics in the K-Channel of Cytochrome c Oxidase

**DOI:** 10.3390/biom12111615

**Published:** 2022-11-01

**Authors:** Rene F. Gorriz, Petra Imhof

**Affiliations:** 1 Department of Physics, Freie Universität Berlin, Arnimallee 14, 14195 Berlin, Germany; 2 Computer Chemistry Center, Friedrich-Alexander Universität (FAU) Erlangen-Nürnberg, Nägelsbachstrasse 25, 91052 Erlangen, Germany

**Keywords:** cytochrome c oxidase, proton transfer, hydration level, molecular dynamics simulations

## Abstract

Cytochrome c oxidase is a membrane protein of the respiratory chain that consumes protons and molecular oxygen to produce water and uses the resulting energy to pump protons across the membrane. Our molecular dynamics simulations with an excess proton located at different positions in one of the proton-conducting channels, the K-channel, show a clear dependence of the number of water molecules inside the channel on the proton position. A higher hydration level facilitates the formation of hydrogen-bonded chains along which proton transfer can occur. However, a sufficiently high hydration level for such proton transport is observed only when the excess proton is located above S365, i.e., the lower third of the channel. From the channel entrance up to this point, proton transport is via water molecules as proton carriers. These hydronium ions move with their surrounding water molecules, up to K362, filling and widening the channel. The conformation of K362 depends on its own protonation state and on the hydration level, suggesting its role to be proton transport from a hydronium ion at the height of K362 to the upper part of the channel via a conformational change. The protonation-dependent conformational dynamics of E101 at the bottom of the channel renders proton transfer via E101 unlikely. Instead, its role is rather that of an amplifier of H96’s proton affinity, suggesting H96 as the initial proton acceptor.

## 1. Introduction

**Figure 1:**
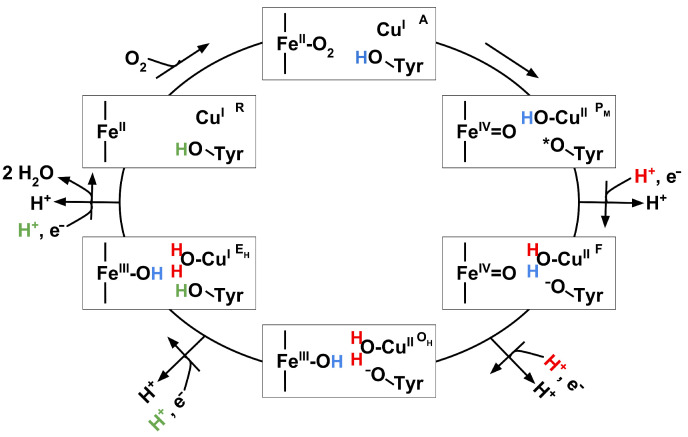
Redox cycle in cytochrome c oxidase. Figure adapted from [22].

Cytochrome c oxidase (CcO), also known as complex IV of the respiratory chain, is a proton pumping membrane protein. It is located in the mitochondrial inner membrane where it uptakes molecular oxygen, electrons and protons. In a redox cycle (see Figure 1), four protons are pumped across the mitochondrial inner membrane. The necessary energy for the pumping mechanism is gained by water formation from oxygen and four “chemical” protons [1,2]. The proton uptake from the N-side to the bi-nuclear centre, the location where redox chemistry takes place, is mediated via two water-filled channels, named D- and K-channels according to essential residues within the channels, D132 and K362 (*R. sphaeroides* numbering). Furthermore, research has shown proton uptake through the K-channel becomes relevant only during the reductive half of the catalytic cycle (states O to R, see Figure 1). Especially in the transition of redox states O→E, the K-channel delivers a chemical proton to the reaction [3,4]. In contrast, both “chemical” and pumped protons pass through the D-channel in the oxidative phase (states A to F in Figure 1) [3].

The crystal structure of *R. sphaeroides* CcO clearly shows a wire of water molecules inside the D-Channel [5], and simulations support water chains allowing proton transfer. Moreover, a self-regulating mechanism controlling the hydration level was observed [6,7,8]. The amount of water present in the D-channel and thus its ability to form water wires is regulated by an interplay of the “asparagine gate”, formed by residues N121 and N139, and the terminal residues D132 and E286 of the channel. The passage of a proton through the “asparagine gate” via a chain of hydrogen-bonded water molecules has been confirmed by simulation studies [7,8] as the rate-determining step of proton transport through the D-channel. Particularly, the protonation state of E286 shows a major impact on the conformation of the “asparagine gate” and thus the channel regulation [6].

In contrast, the number of crystal water molecules found in the K-channel of CcO is too low to enable a purely water-mediated proton transfer. It has therefore been argued that the name-giving lysine K362 may act like a lever elevating the proton by flipping from a downwards to an upwards position [9] (where “downwards” means towards the channel entrance at the N-side of the membrane, and “upwards” refers to towards the bi-nuclear centre and thus towards the P-side of the membrane). Furthermore, mutation studies emphasise the importance of K362 [10,11], and various simulation studies feature a significant probability of K362 to carry a proton [9,12]. Other important titratable residues in the K-channel of CcO, H96 and E101, are located at the channel entrance. The solvent-exposed H96 can occur in neutral and protonated form and would change between these two forms when participating in proton uptake into the channel. E101, which has been discussed as the initial proton acceptor, can also exist in protonated and unprotonated form according to pKa calculations [12].

In case of protonation of K362, proton transfer to Y288, the end of the K-channel, becomes feasible [13,14]. In the presence of an excess charge, the tertiary structure around the K-channel has been observed to undergo conformational changes, including a widening of the channel [9], favouring or disfavouring a change in the hydration within the channel [13,15].

An increased hydration level would allow the excess proton to travel as hydronium ion within a sphere of water molecules, implying that the water cloud rearranges upon the proton transfer between two adjacent water molecules, such that there is always a “leading” water molecule, i.e., a water molecule ahead of the hydronium ion [16]. Moreover, a Grotthuss mechanism along a water wire up to K362 would be conceivable. However, whether such wetting effects indeed occur and thus enable proton transport in the K-channel of CcO still remains unclear.

Hydration levels in the cavities of the BNC and in the proton-conducting channels in CcO have been studied by prediction of internal water sites and molecular dynamics simulations [17,18,19]. The formation of water wires or water chains, along which a proton transport can also take place in the channels [6,14,20] or from the BNC to the P-side and thus the exterior of the protein has been probed by simulations [21]. Higher hydration levels indeed suggest higher connectivity along water chains. In all these studies [18,20], one fixed protonation state of the channel residues has been simulated. and while an effect of the hydration on connectivity by water chains could be observed, the effect of different locations of the excess proton in the K-channel has, to the best of our knowledge, not been studied, yet.

In this paper, we address this question with molecular dynamics simulations of CcO in the O→E redox state, that is, the first transition of a proton through the K-channel in the reductive phase of the catalytic cycle. By varying the position of the (excess) proton in the channel and analysing the hydration pattern, the hydrogen-bonded networks and the conformational dynamics of the protein residues, we find a delicate interplay between hydration and protonation dynamics.

## 2. Materials and Methods

### 2.1. Molecular Dynamics Simulations

#### Model Setup

The model setup is the same as used previously for our molecular dynamics simulations of the Pr→F redox state [22] and of the O→E state [14]. The redox state was modelled as in ref. [9], with a hydroxide ion bound to the Fe(III) of the heme, a water molecule coordinated to the Cu(II) of the BNC, and the terminal residue of the K-channel, Y288 in the deprotonated state (resembling state O${}_{H}$ in Figure 1). Y288 was also cross-linked to H284 (see ref. [9]). The D-channel was modelled with an unprotonated D132 and a protonated E286, representing a situation in which one (to-be-pumped) proton has already passed the D-channel.

In order to explore the impact of the location of the excess proton in the K-channel, we performed simulations in which we varied the protonation states of residues K362, H96 and E101. Of the latter two, either H96 or E101 was protonated, whereas K362 was modelled protonated or unprotonated, with either protonation state of the other protein residues H96 and E101. Furthermore, we performed six additional simulations in which the excess proton was bound to a water molecule, modelled as a hydronium ion (parameters for the hydronium ion are taken from Ref. [23]). To this end, we used the positions of crystal water molecules which are located as listed below (values in parenthesis are the distances of the oxygen atom of the H${}_{3}$O${}^{+}$ ions to the Cα atom of Y288 (in Å)):

(a) Between H96 and E101 (23.4);
(b) Between E101 and S365 (19.1);
(c) Between S365 and K362 (15.5);
(d) Just below K362 (12.8);
(e) Between K362 and T359 (10.4);
(f) Between T359 and Y288 (7.3).

An exception is the hydronium ion between S365 and K362 whose position was modelled by moving the H${}_{3}$O${}^{+}$ ion originally located between E101 and S365 further towards K362. Hydrogen atoms were placed using the “hbuild” tool in the Charmm programme.

In all these simulations, the oxygen atom of the hydronium ions was kept stationary, together with the Cα atom of Y288, to also maintain the relative height of the hydronium ion in the channel. The hydrogen atoms of the hydronium ions were allowed to move to allow them to re-orient according to the dynamic environment.

The resulting protonation possibilities are indicated by a binary code, where 0 means unprotonated and 1 means protonated, respectively. The first four digits have the same meaning as in our previous work [6,22]. The two residues of the D-channel are thus always represented as 01 (Asp132 unprotonated and E286 protonated). The following two digits refer to K362 and E101 in the K-channel, respectively. As an example, model 0110 refers to a model with protonated K362 and unprotonated E101. Models in which H96 is protonated are labelled with an additional “H” such that 0110H refers to a model in which K362 and H96 are protonated.

Models with the proton located on a water molecule (as hydronium ion) are labelled 0100x where x = “a” to “f”, depending on the position of the hydronium ion. K362 and E101 are unprotonated in those models. For example, 0100b refers to a model in which the hydronium ion is placed between E101 and S365. The protonation models studied in this work and their protonation code are listed in Table 1.

The core enzyme complex (subunits I and II) was embedded in a lipid bilayer of phosphatidylcholines and solvated in TIP3 water [24] (25,882 water molecules, ∼115,000 atoms in total). Na${}^{+}$ counter-ions were added by random substitution of water oxygen atoms to neutralize the charge of the system. For protein residues, the CHARMM22 force field [25] was applied, while the parameters for the cofactors were based on quantum chemically derived atomic partial charges and optimized cofactor geometry by Woelke et al. [9]. Parameters for the lipid bilayer were obtained from the CHARMM36 extension for lipids [26]. Simulations were performed using periodic boundary conditions in a tetragonal box of size (x = y = 96 Å, z = 124 Å). Long-range electrostatic interactions were treated using the particle mesh Ewald method [27] on a 96 × 96 × 128 charge grid. A non-bonded cutoff of 12 Å was applied. The short range electrostatics and van der Waals interactions were truncated at 12 Å using a switch function starting at 10 Å.

### 2.2. Molecular Dynamics Simulations

After minimising the systems for 5000 steps by steepest descent and heating for 30 ps to 300 K, three stages of equilibration (with decreasing harmonic restraints on the solute atoms) were performed in which the numbers of particles, pressure (1 bar) and temperature (300 K) were kept constant (NPT ensemble) during 75 ps. Pressure control was introduced using the Nosé-Hoover Langevin piston with a decay period of 500 fs. Then, three replicas of 200 ns long NPT production runs with 2 fs integration time step (using a velocity-verlet integrator), started with different initial velocities, were performed, and coordinates were saved every 2 ps. All covalent bond lengths involving hydrogen atoms were fixed using the SHAKE algorithm [28].

These molecular dynamics (MD) simulations were carried out using the program NAMD2.14 [29].

### 2.3. Analysis

The first 40 ns of the simulation time was regarded as further equilibration, similar to our previous work [14,22], thus leaving the last 160 ns for analysis. All properties were evaluated for each of the three independent simulations separately and then averaged. The standard error from this mean is used for error estimates.

All analyses were performed using our own Python scripts. In the case of the hydrogen-bond lifetimes, this is an adaption of the respective code in MDanalysis [30] and Java-written code, which uses the JGraph library [31]. Molecule figures were generated with vmd [32], and all plots were generated with matplotlib in Python [33].

#### 2.3.1. Channel Hydration

**Figure 2:**
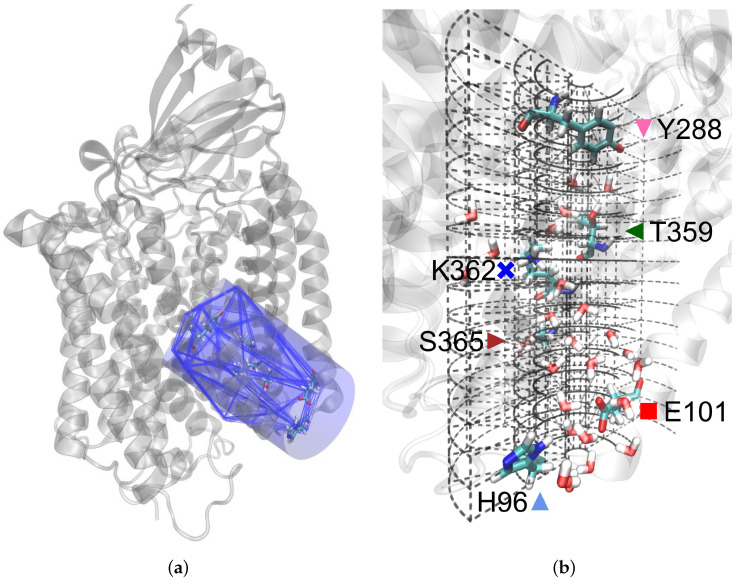
(**a**) Polyhedron of the K-channel drawn every 16 ns enveloped by the cylinder; (**b**) segmentation of the cylinder.

To identify water molecules that interact with the K-channel, we defined the surface of the channel by a polyhedron. Its corners were given by the centres of masses of the residues L227, L238, H284, V287, P315, G324, G327, A356, I363, S365, W366 and G398 of chain A and H96, L100 and E101 of chain B. For every time frame in the trajectory, the volume maximised polyhedron was calculated (see Figure 2a). Each water molecule, determined by the position of its oxygen atom, which hit the polyhedron volume at least once was considered for further evaluations.

For spatial visualisations within the channel, the polyhedron was enveloped by a cylinder of radius 9.5 Å such that all water molecules which were identified to be within the polyhedron were also captured inside the cylinder. To construct the cylinder, the frame closest to the average atom positions (representing a “median structure”) was used. This frame was calculated for each trajectory from the first 10 ns analysed.

The cylinder axis was formed between the centres of masses of the C${}_{\alpha }$ atoms of the lowermost and uppermost residues, respectively. For proper coverage of the entrance and exit of the channel the axis was elongated by 10%. Its resulting maximum height was 30 Å. Along the axis, the cylinder height was partitioned in 15 slices of 2 Å thickness each. The thickness of a slice was chosen to be larger than the largest distance (the H–H distance is ∼1.5 Å) in a (TIP3P) water molecule, but by about the same amount smaller than the H⋯O distance in a hydrogen bond (∼2.5 Å) to a neighbouring water molecule. The slices were again subdivided by the distance to the axis, resulting in five radial sections within a slice by the radii r ≤ 1.0 Å, 1.0 Å < r ≤ 3.5 Å, 3.5 Å < r ≤ 5.0 Å, 5.0 and the rest 5.0 Å< r ≤ 9.5 Å. Furthermore, each ring had a radial resolution of 30${}^{\circ }$ segments (see Figure 2b).

#### 2.3.2. Water Mobility

For each slice in the cylinder, *s*, diffusion coefficients, $D_{s}$, were calculated from the distances travelled by a water molecule, *w*, inside this slice per time as mean squared displacement

(1) $$D_{s}=\frac{{\langle \frac{1}{N_{s}}\sum_{w}{\left(x_{w,s}\left(t\right)-x_{w,s}\left(t-\tau \right)\right)}^{2}\rangle }_{t}}{6\tau }$$

where τ = 2 ps, and $N_{s}$ is the total number of water molecules observed in the slice *s* over the simulation time analysed. Mobilities of water molecules leaving a slice in one time frame were assigned to the starting slice. Note that the probability to travel further than one slice in one frame is less than 0.25%.

The reported values are averages, weighted by $N_{s}$, over the three simulation runs, respectively. Errors were estimated as the standard deviation of this average. For slices with insufficient data, namely empty slices in more than 95% of the time, the diffusion coefficient is given as zero.

#### 2.3.3. Conformational Analysis

We analysed the side chain conformations, i.e., dihedral angles $\chi_{1}$ to $\chi_{4}$ (where applicable) of protein residues Y288, S299, T359, K362, S365, H96 and E101 as well as the distances between some of these residues. For the distance analysis, we considered the polar atoms of the side chains, i.e., oxygen atoms in tyrosine, serine and threonine residues and the nitrogen atom Nζ for K362, respectively. For H96, we used atom Cϵ1, to which the two nitrogen atoms of the side chain are bound, and for E101, we used atom Cδ, to which the two oxygen atoms of the side chain are bound. We will refer to an “up” conformation if a residue’s side chain is pointing towards the BNC or the P-side of the membrane and a “down” conformation when such a side chain points towards the N-side of the membrane.

Furthermore, the distance between Cα atoms of P358 and A319, M316 and K362, P315 and E101, and P315 and S365 were analysed as a measure for the channel width. The two former were used previously in ref. [9]; the latter two are introduced as a measure for the channel width in the lower region.

#### 2.3.4. Hydrogen Bond Probabilities and Lifetimes

We analysed hydrogen bonds between the K-channel residues Y288, T359, K362, S365, H96 and E101 and between those protein residues and water molecules. The existence of a hydrogen bond is defined by the geometric criterion of a cutoff of 3.5 Å for the donor–acceptor distance and a deviation of 35 degree from linear for the donor–hydrogen–acceptor (D-H⋯A) angle.

Hydrogen bond lifetimes were calculated between important protein residues and water molecules via the time auto-correlation function using the following equation (Equation (2)):

(2) $$C\left(\tau \right)=\langle \frac{\sum h\left(t_{0}\right)h\left(t_{0}+\tau \right)}{\sum h{\left(t_{0}\right)}^{2}}\rangle$$

where *h* is a binary measure of whether a hydrogen bond exists, h=1, or if not, h=0, irrespective of the identity of the water molecule, $h(t_{0})=1$ indicates a hydrogen bond at a time $t_{0}$, and $h(t_{0}+\tau )=1$ indicates that the protein residue remains hydrogen-bonded to a water molecule throughout the period $t_{0}$ to $t_{0}+\tau$. Note that the individual water molecule as well as the individual donor, acceptor and hydrogen atoms can change. The hydrogen bond lifetime is obtained by fitting the time auto-correlation curve with a bi-exponential function:

(3) $$C\left(t\right)=A\cdot exp\left(-t/\tau_{1}\right)+B\cdot exp\left(-t/\tau_{2}\right)$$

where $\tau_{1}$ and $\tau_{2}$ represent two time constants, one corresponding to a short-timescale process and the other to a longer timescale process. Amplitudes *A* and *B*, which add up to 1, represent the respective weights of the short- and longer-timescale processes in the overall autocorrelation curve [30]. The reported hydrogen bond lifetimes are the weighted sums of the time constants of the two processes $\tau =A\cdot \tau_{1}+B\cdot \tau_{2}$.

We evaluated the auto-correlation up to a maximal lag time τ=100 ps. To improve statistics, multiple time origins, $t_{0}$, were used in the calculation, and the average was taken over all time origins. The period between time origins, $t_{0}$, was chosen as 120 ps such that the individual auto-correlation sequences do not overlap.

Hydrogen-bond connections between protein residues inside the K-channel, i.e., Y288, T359, K362, S365, H96 and E101, could be formed directly or via water molecules. To find the probabilities for such water-mediated hydrogen-bond connections, for each time frame a graph was set up in which the protein residues and water molecules represent the nodes and edges represent existing hydrogen bonds between a pair of residues in that frame. On that graph, a shortest path search using Dijkstra’s algorithm [34] between two nodes representing protein residues was performed. If such a path exists, the hydrogen-bonded connection between the two residues considered is counted.

## 3. Results

**Figure 3:**
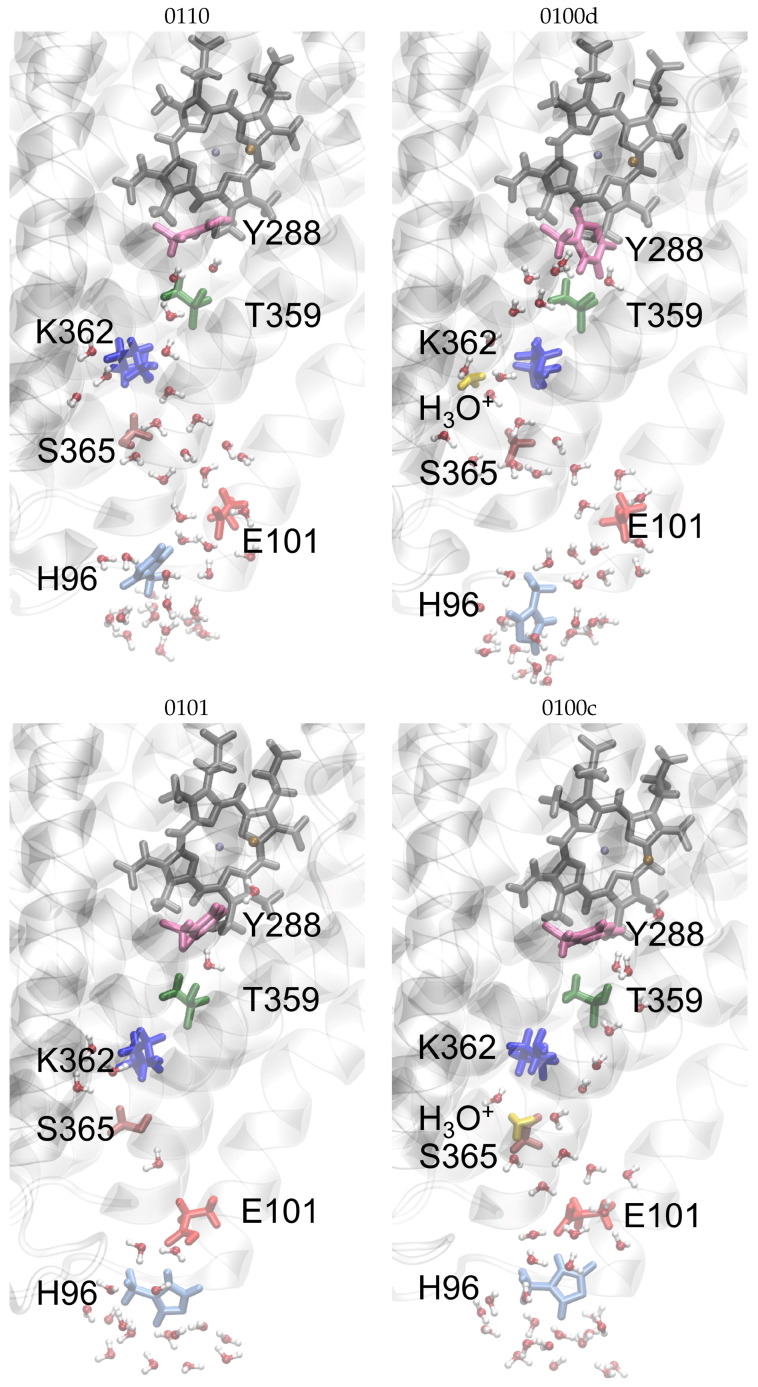
Median structures of the K-channel of CcO from the MD simulations of the most important different protonation states. For the other models, see Figure S1 in the Supplementary Material. Important protein residues are marked by their labels and colours: H96 (light blue), E101 (red), S365 (brown), K362 (blue), T359 (dark green), Y288 (magenta) and the H${}_{3}$O${}^{+}$ ion (yellow), if present.

Figure 3 and Figure S1 show snapshots, representing median structures of the MD simulations, of the K-channel of CcO in the different protonation states simulated in this work.

### 3.1. Channel Hydration

As can be seen in Table 2, the number of water molecules inside the K channel of CcO varies considerably with the position of the excess proton. This variance is pronounced differently when considering only water molecules in the polyhedron, defining the K-channel, or all water molecules in the enveloping cylinder. The volume of the polyhedron also varies significantly between the different models, indicating conformational variances of the protein residues defining the polyhedron. A qualitative inspection of the trajectories shows that there is, moreover, an exchange between water molecules inside the channel and at the surface at the channel entrance. The volume of the polyhedron correlates well with the average number of water molecules inside this polyhedron. The additional water molecules observed in models with high hydration level are former surface or bulk water molecules.

Models with a protonated K362 (but unprotonated E101) exhibit a significantly higher hydration than models in which K362 is neutral. As an extreme, compare 0110 and 0100 with a hydration difference by a factor of about six. The effect is, however, reduced to 2–3 fold when H96 or E101 are also protonated (compare 0100H and 0110H, and compare 0101 and 0111). The hydration level in models with a hydronium ion increases with the height of the hydronium ion, from the lower regions 0100a, 0100b and 0100c, up to a maximal value (among models with hydronium ions) in the 0100d model, in which the proton is located close to K362, and then a further reduction occurs when the hydronium ion is past K362, models 0100e and 0100f, with hardly any water molecules between S365 and K362 in the latter. These findings confirm that, up to the height of K362, a hydronium ion inside the K-channel of CcO can lead to more and more water molecules entering the channel and can thus provide its own water environment. It is interesting to note that models 0110 and 0110H with protonated K362 exhibit the highest hydration level and also the largest volume of the polyhedron.

**Figure 4:**
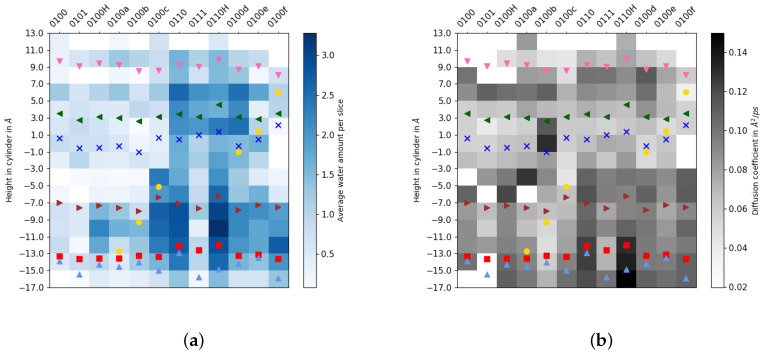
(**a**) Channel hydration and (**b**) water mobility, discretised by cylinder height for different protonation models of the K-channel of CcO. The symbols mark the average height of protein residues H96 (light blue triangle 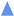), E101 (red square 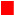), S365 (brown triangle-right 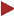), K362 (blue cross 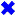), T359 (green triangle-left 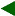), Y288 (magenta triangle-down 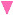) and position of the H${}_{3}$O${}^{+}$ ion (yellow circle 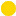), if present.

**Figure 5:**
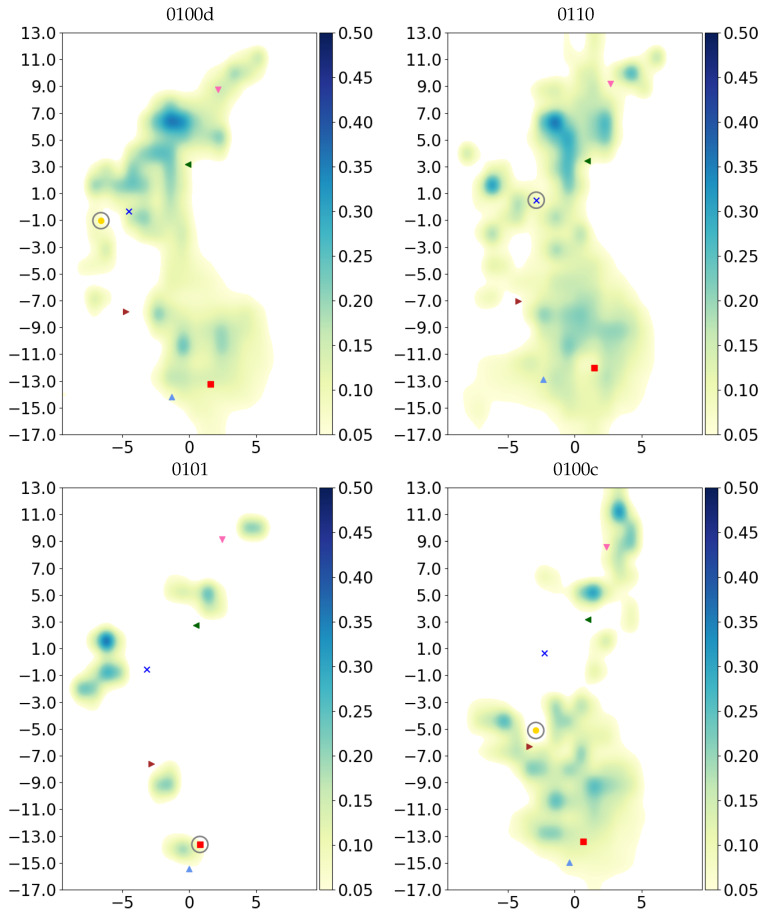
Projection of water occupancy in the K-channel of CcO with most important protonation states. For the other models, see Figure S3 in the Supplementary Material. The symbols mark the average height of protein residues H96 (light blue triangle 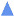), E101 (red square 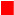), S365 (brown triangle-right 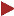), K362 (blue cross 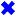), T359 (green triangle-left 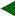), Y288 (magenta triangle-down 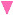) and position of the H${}_{3}$O${}^{+}$ ion (yellow circle 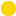), if present. Residues with an excess proton are marked by a grey circle around the symbols representing the respective residues.

Further analysing the height (as a projection onto the cylinder axis) dependence of the hydration level (see Figure 4) and as 2D projection (Figure 5), one can see that only in the more hydrated models water molecules are observed between S365 and K362. The region between E101 and S365 shows a rather low number of molecules in models with protonated E101, even for model 0111 that has a total number of water molecules in the K-channel that is comparable to that of model 0100c. In the latter, however, the water molecules are predominantly located in the lower part of the channel, whereas in 0111, the upper part is more hydrated.

Inspecting the accumulated number of water molecules up to a certain height in the channel (Table S2 in the SI), this number remains constant between the height of S365 and K362 in the models with fewer water molecules in total, that is, models 0100, 0101, 0100H, 0100a and 0100b with the excess proton located below S365. Once the excess proton has reached S365, or is above this residue, the amount of water molecules continues to rise also between the heights of S365 and K362, “slowly” filling up the channel, albeit with a low total number of water molecules in model 0111. An exception is model 0100f in which the excess proton has almost reached Y288 at the end of the channel.

Models with water molecules stretching all the way from the channel entrance via K362 to Y288 at the top have either K362 protonated or a hydronium ion located at the height of K362 or higher.

Analysis of the water mobility (see Figure 4b) reveals that water molecules at a height just below K362 (∼−2 Å) up to T359 are rather immobile, whereas they exhibit significant diffusivity between T359 and Y288 (as long as they are present), most of which is at the same height (in angular direction and to/from the cylinder axis cf. Figure S2 in the SI). Water mobility is highest in the lowest part of the channel, i.e., up to about the height of E101 (∼−13–−12 Å) and in the more hydrated models up to S365 (∼−7 Å). Some models show a very high water mobility at heights with very little water density, e.g., models 0100H and 0100a above S365 and model 0100b just above K362. This suggests that any water molecule reaching such height leaves it again quickly. According to the different contributions to the diffusivity (see Figure S2 in the SI), this movement is observed along the transmembrane direction (i.e., along the cylinder axis). Judging from the low occupancy of water molecules at the cylinder slice above S365 (Figure 4a) and the generally lower hydration level in the upper part of the channel in models 0100, 0100H, and 0100a, these highly mobile water molecules likely “return” quickly to the lower part of the channel and do not proceed to the upper part.

### 3.2. Conformational Analysis

#### 3.2.1. Side Chains of Protein Residues

The conformational dynamics of the side chains of the protein residues, as described by the probability distribution of dihedral angles $\chi_{1}$ and $\chi_{2}$, are not much affected for side chains Y288, T359 and S365 (see Figures S4–S7 in the SI). Y288 exhibits a second $\chi_{2}$ state in models 0100d and 0100f which have a hydronium ion in the upper region. T359 does so in the 0100f model. Residue S365 shows a second state for $\chi_{2}$ in the models 0110 and 0110H, i.e., with protonated K362, and in the 0100d model (see Figure S6). For all models with the excess proton below S365, i.e., 0100, 0101, 0100H, 0100a and 0100b, the $\chi_{1}$ angle value of S365 is exclusively at $\approx -60^{\circ }$ and the $\chi_{2}$ angle value at $\approx +60^{\circ }$, whereas the other models also show conformations with $\chi_{1}\approx 180^{\circ }$ and $\chi_{2}\approx 180^{\circ }$. In model 0100c, which has the hydronium ion placed in close vicinity of S365, this residue has a second state with $\chi_{2}$ angle of $\approx -60^{\circ }$ that is not observed in any of the other models (see Figure S6).

**Figure 6:**
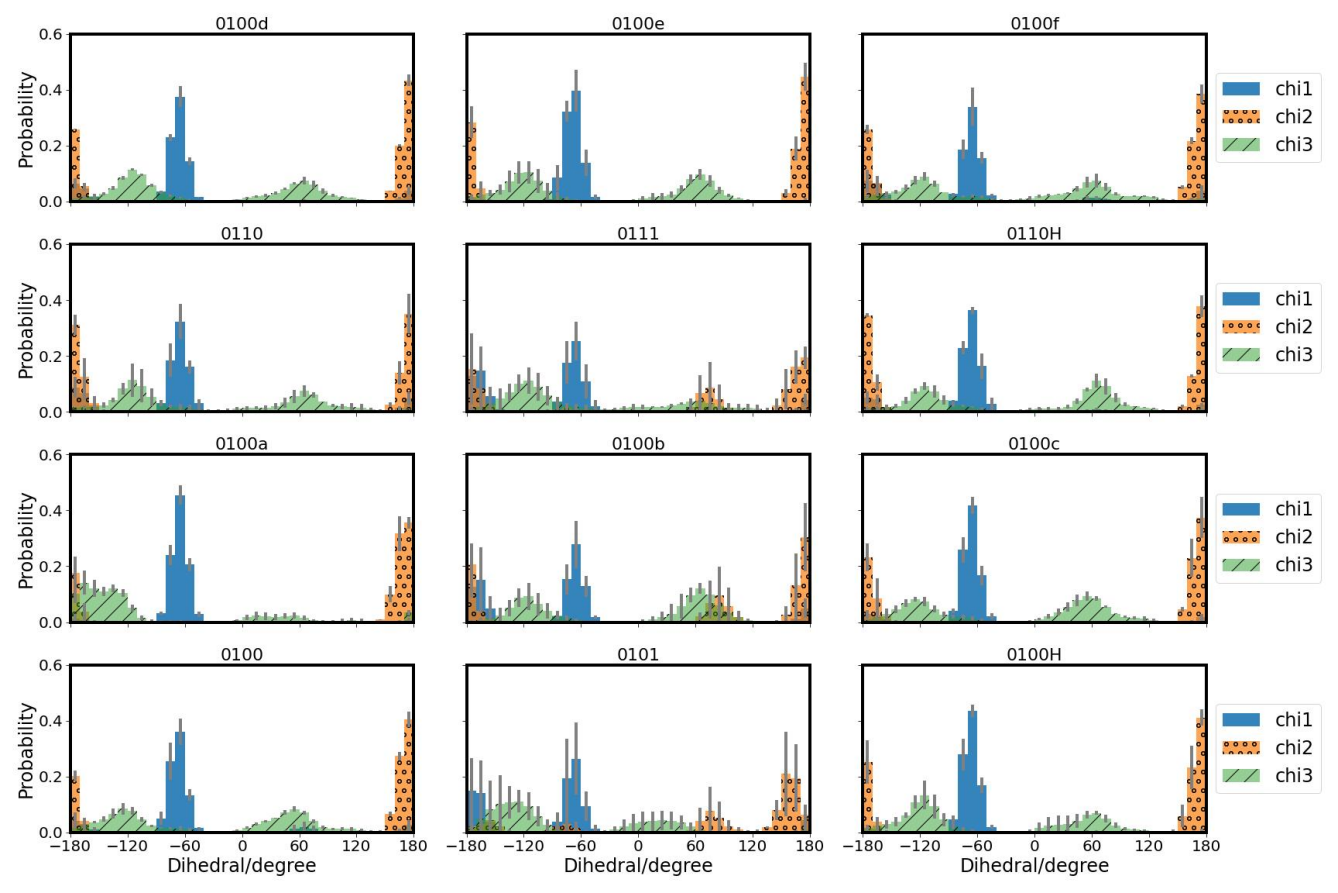
Probability distributions of side chain dihedral angles of E101 in the K-channel of CcO, modelled in different protonation states.

The conformation of residue E101 is mainly influenced by the residue’s own protonation state. Models with protonated E101 (0101 and 0111) populate one $\chi_{2}$ angle state more than the other models (see Figure 6 and Figure S13). This dihedral state is also observed in model 0100b which has the hydronium ion located above E101 and close to S365. Inspection of the distances between E101 and Y288 reveal that for the models with protonated E101 this conformation corresponds to a shorter distance to Y288 and for model 0100b to a shorter distance to T359, i.e., the side chain of E101 points “up” towards Y288 or T359, respectively (Table 3 and Table S3). As opposed to the other models, E101 hardly populates a conformation with $\chi_{3}∼60^{\circ }$ when protonated or when the hydronium ion is below this residue (0100a and 0100b) (see Figure 6 and Figure S14).

H96 varies mainly in the probability of essentially two states defined by its $\chi_{1}$ angle (−60${}^{\circ }$ or +60${}^{\circ }$). Only in the 0100 model, is another state with $\chi_{1}\approx \pm 180^{\circ }$ observed. According to the distances between H96 and Y288 (Table 3), the histidine residue can populate several states that correspond to distances to Y288 between ∼25 and ∼30 Å.

**Figure 7:**
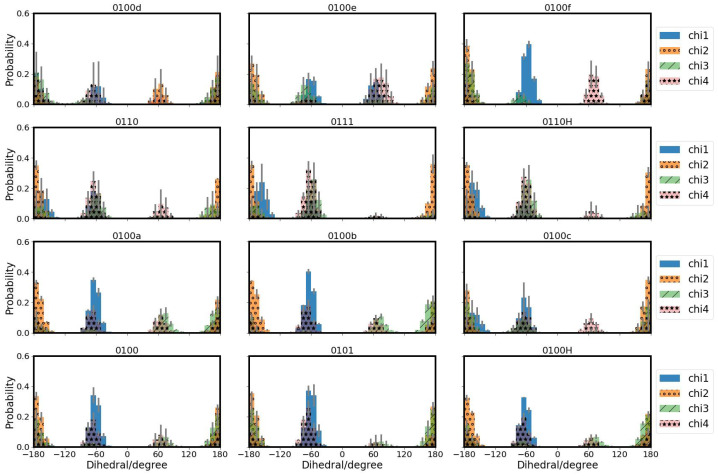
Probability distributions of side chain dihedral angles of K362 in the K-channel of CcO, modelled in different protonation states.

K362 has the shortest average distances to Y288 when it is protonated or with a hydronium ion located above K362 (0100e and 0100f). With a hydronium ion between S365 and K362 in model 0110c, K362 exhibits a short average distance to Y288 (see Table 3). The same holds for the distances to T359 (see Table S3) which is also above K362. Accordingly, the $\chi_{1}$ and $\chi_{3}$ distributions of model 0100c resemble more those of models with protonated K362 (see Figure 7).

Together, this shows a preference for an “up” conformation of K362 when a proton is located at K362 or above this residue.

#### 3.2.2. Channel Size

Comparison of the size of the K-channel for the different protonation models reveals a correlation between the hydration level and the channel volume. Models with protonated K362 (0110, 0110H) and 0100d that have a high average number of water molecules (17–19) inside the channel are also those models in which the channel has a larger volume. Models 0100c, 0111 and 0100e with intermediate number of water molecules (10–12) exhibit a lower volume, and models 0100 to 0100b have an even lower volume and also an even lower average number of water molecules inside the channel (Table 2).

Comparing the widths of the K-channel measured at the height of K362 (via distances between P358-A319 and M316-K362, Table 3), again shows a wider K-channel for models with protonated K362 (except for 0111) and models 0100c and 0100d. Model 0111 exhibits distances representing the channel width that are shorter than in the other models with protonated K362 but longer than the other models with comparable hydration level (0100c and 0100e).

The P315-E101 distance in model 0111, however, corresponds to a channel width at the height of E101 that is similar to that of the lower hydrated models. This suggests that in model 0111, although the central and upper part of the channel are large and wide enough to accommodate more water molecules, the channel is too narrow at the height of E101 to allow more water molecules to enter and move further up. In this particular model, the narrow width is caused by E101 that can also be in an “up” conformation (corresponding to a shorter distance to Y288, and $\chi_{1}$ and $\chi_{2}$ angles that are not observed otherwise, see Figures S12, S13 and S17 in the SI). The same is observed in model 0101 which also has E101 protonated in an “up” conformation and is the model with the lowest number of water molecules inside the channel. To some extent, this also holds for model 0100c which has the proton just above E101. There is also a narrow channel width at the height of E101, some probability for E101 to be in an “up” conformations, and a low number of water molecules in the channel.

Further comparing the models with neutral K362 and unprotonated E101 to those with a hydronium ion in the lower part of the channel (0100a and 0100b), the lower channel volume of those models is reflected in the shorter distances used to measure the channel width. In model 0100b, the P315-E101 distance is almost as short as in models with protonated E101, in agreement with the “up” conformation of E101 (Table 3).

In all these models, K362 is in a “down” position, corresponding to larger distances to Y288 and T359 (cf. Table 3 and Figures S19 and S21 in the SI). In model 0100d, which has a hydronium ion next to K362, the conformation of K362 is also “down”, as manifested by the distances to Y288 (Table 3). K362 is, moreover, in a different torsional state, as shown by $\chi_{1}$ and $\chi_{2}$ angles (see Figure 7, Figures S8 and S9 in the SI).

This particular K362 conformation is observed only in model 0100d, the only highly hydrated model with unprotonated K362. For all other models, K362 in an “up” conformation correlates with a larger channel width at the height of K362.

In model 0100c, K362 exhibits a rather short distance to Y288, indicating an “up” conformation, in line with the dihedral angles of K362 resembling those of models with protonated K362. This model has an intermediate hydration level and an intermediate P358-A319 distance, but otherwise distances that correspond to a wider channel (Table 3).

The channel width at the height of S365, as measured by the P315-S365 distance (Table 3) is larger for models with protonated K362 or model 0100d with the hydronium ion at the height of K362, i.e., the models with the highest hydration level. Model 0100c, in which the hydronium ion is located above S365 but not at K362 (nor above) has a channel width at the height of S365 that is comparable to the models with lower hydration level.

### 3.3. Hydrogen Bonds

The average number of hydrogen bonds between protein residues and water molecules (see Table 4) follows the trend of the hydration level: models with protonated K362 and 0100d that have more water inside the channel are also more likely to form hydrogen bonds between protein residues and water molecules. The average number of hydrogen bonds to water of residue K362 increases by more than one when this residue is protonated, indicating that not only the additional hydrogen atom contributes to another hydrogen bond with K362 but also the charged residue is more likely to form hydrogen bonds with water than its neutral counterpart. That the presence of the excess charge is even more important than the number of water molecules available is further supported by the 0100d model, which has a high hydration level but a probability for K362 and for S365 to form hydrogen bonds with water that is comparable to other models with neutral K362. For the hydronium ion itself, about one more hydrogen bond with water molecules is observed in models 0110c and 0100d with high hydration levels than in models with lower hydration levels.

Another extreme case in which the number of hydrogen bonds clearly depends on the residue’s protonation state is E101. In its neutral form, it has on average 1.5 hydrogen bonds, whereas a charged E101 reaches up to to 4 hydrogen bonds on average.

It is also this residue, E101, that shows extreme behaviour in its hydrogen bond lifetimes. These are either several hundreds of picoseconds, according to the bi-exponential fit of the autocorrelation time functions, or cannot be determined due to too large errors. For all other residues, the hydrogen bond lifetimes are longer the higher the probability for a hydrogen bond is, and the higher the number of water molecules inside the channel is, which can be expected. An extreme case is K362 which, similar to E101, has very long hydrogen bond lifetimes (∼1 ns) when it is charged but only on the order of 10–20 ps in its neutral form (see Table 5). Similarly, the lifetime for hydrogen bonds between S365 and water molecules is longer when there are more water molecules present, below and above S365. H96 is an exception in that it exhibits significantly longer lifetimes of hydrogen bonds with water molecules when protonated, although the average number of hydrogen bonds is not larger than in models with neutral H96.

**Figure 8:**
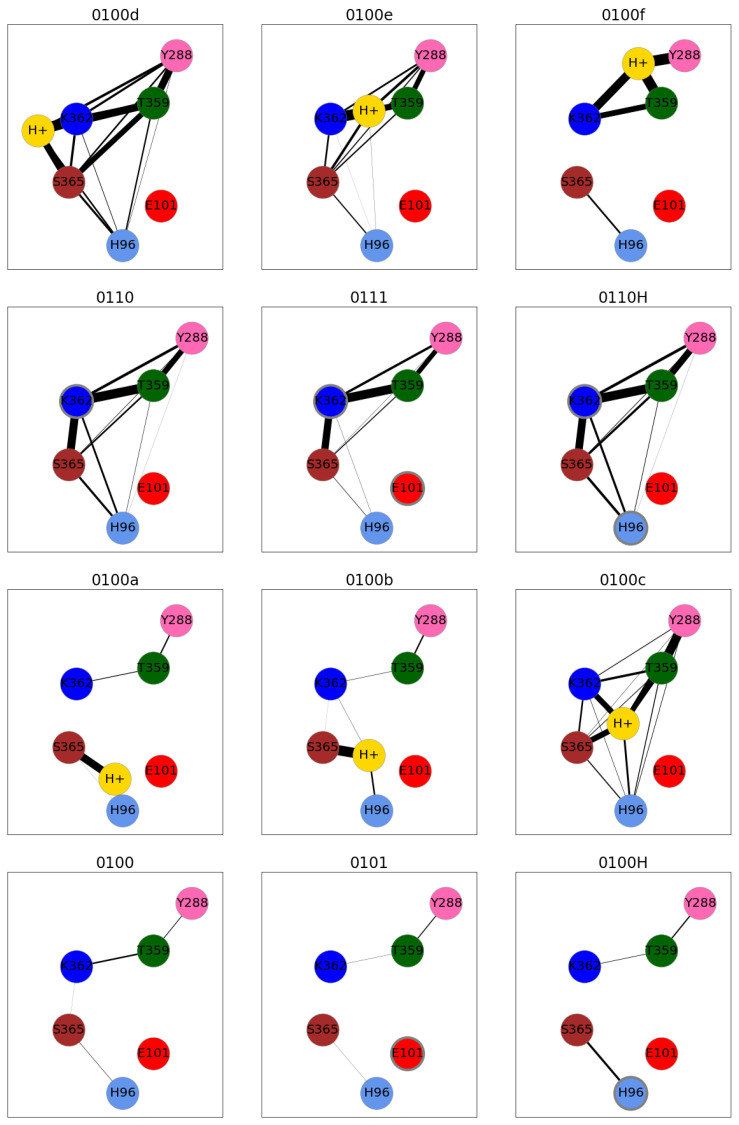
Networks of hydrogen-bonded connections between protein residues in the K-channel of CcO, modelled in different protonation states. Residues are represented by coloured circles (magenta: Y288, green: T359, blue: K362, brown: S365, light blue: H96, red: E101). Grey circles mark protonated residues. The thickness of the connecting lines corresponds to the probability of a hydrogen-bonded connection to be present.

The formation of hydrogen bonds between protein residues is a first prerequisite for hydrogen-bonded chains along which proton transfer can occur. The probability for such chains, which can span several water molecules, is depicted as hydrogen-bonded networks in Figure 8 where thick lines between protein residues (coloured circles) correspond to high probabilities for hydrogen-bonded chains between the residues. The highest connectivity in such a network is observed for the models with high hydration level, that is, those with protonated K362, 0100c and 0100d. Model 0111, also having a lower hydration level than the other aforementioned models (see Table 2), has a probability for hydrogen-bonded connections between S365 and K362, and up the channel to T359 or Y288, which is comparable to that in the highly hydrated models 0110, 0110H and 0100d (see Figure 8). This indicates that a proton transfer from S365 all the way up the channel is conceivable. In models with the excess proton in the lower part of the channel, 0101, 0100H and 0100a, there is no hydrogen-bonded connection between the lower part, H96 or E101, and S365 and the upper part, K362, T359 and Y288. This corresponds to the low number of water molecules in the region between S365 and K362 in those models. Such a connection would be necessary to reach K362 and from there allowing proton transfer further up. In model 0100b, there is a high probability for a hydrogen-bonded connection between S365 and the hydronium ion, and also a hydrogen-bonded connection between the hydronium ion and K362 can be formed, albeit with low probability. Once the excess proton is above S365, as exemplified by model 0100c, hydrogen-bonded connections between the lower and upper part of the channel have a significant probability, rendering proton transfer through the channel feasible.

## 4. Discussion

Our molecular dynamics simulations of CcO with an excess proton located at different positions in the K-channel, either on protein residues or as hydronium ions, afforded us the chance to explore the dependence of the hydration level of the K-channel on the proton position.

The probabilities of hydrogen-bonded connections and the lifetimes of hydrogen bonds, along with the probability for a proton transfer to take place along hydrogen-bonded connections, highly correlate with the number of water molecules in the channel. According to our simulation data, proton transfer across the channel is likely when the proton is located above S365 (model 0100c), at about the height of K362 (model 0100d) or when K362 is protonated but E101 is not (models 0110 and 0110H). These are also the models with the largest volume and highest number of water molecules inside the channel.

Models in which the proton-carrying water molecules, i.e., the hydronium ion, are in the lowest part of the channel (models 0100a and 0100b) exhibit a rather low hydration level, with particularly few water molecules in the upper part (above S365) of the channel. Located in the lower part of the channel, the hydronium ion drags its hydration cloud only as far as it has reached itself. As our projection of the water density shows, there is little probability density for a water molecule ahead of the hydronium ion (except for the positions of crystal water molecules). This further suggests that the presence of a positive charge alone is not sufficient to fill the channel with water, and the attraction of further water molecules dragged by the proton is limited to the immediate hydration cloud.

Comparison of models 0100b, 0100c and 0100d in which a hydronium ion works its way up the channel (from below S365, to above S365, and to the height of K362) and drags a water cloud with it, suggests the hydration level becomes high before the proton is passed on to K362 and remains high with the proton at K362 (model 0110). Models with a hydronium ion above K362 (0100e and 0100f) show a well-connected upper part of the K-channel but lower hydration level than models with protonated K362 or those with the hydronium ion further below, 0100c and 0100d. The lower hydration level in models 0100e and 0100f can be explained as the excess charge being located too far away from the channel entrance to attract water molecules from the bulk. Moreover, in the course of the simulation of these models, the channel was never wide at the height of K362. It is unlikely that a passage of the excess proton from K362 to the upper part of the channel leads to a loss of water molecules and the channel to “dry”. Rather, water molecules that are already inside the upper part of the K-channel remain, and the actual number is higher than that observed in the simulated models 0100e and 0100f. It is quite possible that the MD simulation time of 200 ns is too short to allow full sampling of major structural changes such as (unfavourable) channel widening and conformational changes of protein residues. Most residues show a clear preference for one or a few dihedral conformations in a particular protonation model. Likewise, different channel widths and hydration levels can be observed for different protonation models, suggesting that the individual models are sufficiently equilibrated to show the interplay of protonation state, local protein conformation and, importantly, proton transport and hydration level in the channel.

The hydration level is highest in the 0110 model, in which K362 points “up”, indicating that this conformation can lead to a wider channel, allowing more water molecules inside. Channel widening as a pre-requisite for a flip of K362 to an “up” position is unlikely since model 0111 shows an “up”-like K362 conformation and a narrow channel. Models 0110H and 0110 with a protonated K362 in “up” conformation, in contrast, exhibit a large channel volume and a higher hydration level. Hence, it appears more reasonable that the higher number of water molecules in 0110 and 0110H accounts for the wider channel, at least to some extent and not the conformation of K362.

The importance of a channel widening to increase the hydration level has been shown by experiments with a fluorescent marker at the channel entrance [15]. When Helix 6 (containing H96) and Helix 8 (containing E101) widen, they give space for water molecules to enter the channel. While this explains how a higher hydration level at the channel entrance can be reached, the cause and consequence of the wider channel, higher channel volume and higher number of water molecules in the K-channel cannot be explained this way. In the lowest part (below S365) of the channel, the channel width is sufficient to accommodate larger numbers of water molecules, or in other words, an increased hydration level in the lower part of the channel does not lead to a larger polyhedron volume and/or widening of the channel. For the part of the channel above S365, the channel widens, and the volume increases with the hydronium ion and its water cloud moving up (as can be envisaged as transitions from models 0100a, 0100b, 0100c to 0100d). This suggests the moving water cloud to be the reason for the channel widening.

When a proton is located on E101, the side chain of E101 has a tendency to point “upward”. Since this is the neutral, or not-negative form, one can argue that it is not repelled by the negatively charged Y288 and can therefore point “up” towards Y288. Since Y288 is rather distant, though (>20Å), it is more likely that the hydrophobic (“dry”) interior of the K-channel is more favourable for E101’s side chain than the solvent-exposed channel entrance. This “up” conformation of E101 narrows the lower part of the channel, as manifested by short distances to P315, preventing (further) water molecules from entering. Correspondingly, even with protonated K362, the channel hydration is rather low when E101 is also protonated (model 0111).

According to mutation experiments [35,36], E101 is essential for the proton transport through the K-channel and has therefore been discussed to be the residue responsible for initial proton uptake. According to our simulations, as long as E101 is protonated, the channel has only a few water molecules inside and is unlikely to fill further. For the proton transport to proceed, the proton must thus be transferred to a nearby water molecule, of which there are so few that such a transport does not go farther than E101, just to a state corresponding to 0100a.

Alternatively, H96 can act as the residue that initially accepts a proton from the bulk. Then, E101 must play another critical role. One such role could be the amplification of H96’s proton affinity, E101 and H96 together forming a “proton acceptor diad”. This idea is supported by the 0100H model showing the shortest distances between the two residues (note that the distances listed in Table 3 are not donor–acceptor distances but measured between the Cϵ1 and the Cδ atom of H96 and E101, respectively).

The protonation model 0100H reflects the scenario of H96 initially accepting a proton from the bulk. This model allows enough water molecules in the lower part of the channel to directly transfer a proton via hydrogen-bonded chains up to S365, or at least to a state corresponding to model 0100b. Though the water density beyond S365 is low in model 0100b, there is a small probability of forming hydrogen-bonded connections between the hydronium ion and K362, as well as between S365 and K362, indicating a (small) chance for water molecules to pass S365. It is therefore conceivable that a state, corresponding to model 0100c can be formed, albeit with low probability. As soon as the excess proton is beyond S365, the hydration level and the hydrogen-bonded connectivity increase, rendering proton transfer through the channel feasible. Passage of S365, first by a water molecule and then by the proton itself, is therefore likely the rate-limiting step of proton transport through the K-channel.

Though even a hydrogen-bonded connection from the hydronium ion to T359, and therefrom to Y288, is observed in models 0100c and 0100d, when the hydronium is past S365, the water distribution still renders a pathway via K362 more probable. The “down” conformation of K362 observed in model 0100c allows proton uptake from the lower part of the channel by K362. In model 0100d, the orientation of K362 represents an intermediate state between a “down” and an “up” conformation that allows K362 to accept a proton from the hydronium ion and possibly subsequent proton donation to water molecules above K362. Reaching such a conformation is likely supported by the increased channel width in this model. Both factors are related, since a lack of water molecules between S365 and K362 does not allow the formation of a hydrogen-bond connection between the two residues and thus the transport of a proton from the lower to the upper part of the channel.

Once the proton has arrived at K362, it points “up” to the negatively charged Y288. In such a conformation, the high hydrogen-bonded connectivity renders proton transfer via a Grotthuss mechanism along hydrogen-bonded chains likely, in agreement with simulation studies of explicit proton transfer [13].

Getting the proton to K362 has been considered as an important step/pre-requisite for proton transport through the K-channel [9,13,37]. While a number of previous simulation studies suggest a water-mediated hydrogen-bonded network between K362 and Y288 [17,18,20,38], partially via T359, regardless of K362’s protonation state, with neutral K362 no connectivity with the lower part of the K-channel has been observed [18,38] but with a protonated K362 there is enough water in the lower part to render water chains between K362 and E101 conceivable [20]. Our simulation data agree in as much as protonated K362 leads to a high connectivity of hydrogen bonds in the upper and lower part of the channel. Moreover, a hydronium ion located above S365 or at the height of K362, can lead to water-mediated hydrogen-bonded networks, “simply” due to the increase of water molecules present.

This increased water level could only be observed because of the (artificial) placement of hydronium ions and a molecular dynamics simulation that allows the protein structure to change such that a channel widening and with that a further filling of the channel can take place. Water insertion methods that work with static structures but also grand canonical Monte Carlo sampling may not be able to fully capture this “filling” of the channel (an increased number of water molecules also in the upper part of the channel) if it requires some channel widening (due to extra water molecules) to allow further water molecules to enter, attracted by an excess proton.

Our data show that the location of the excess proton is decisive for the hydration level. In particular, the region between S365 and K362 remains “dry” unless a hydronium ion is located there. For a hydronium ion to move beyond S365, or the proton to hop over to a water molecule beyond S365, the hydronium ion must have already moved with its hydration cloud up to just below S365, and one water molecule must be above S365. This passage of a water molecule is thus crucial for proton transport up to K362. Once this has been achieved, the channel fills up with sufficient water to facilitate further proton transport up to Y288 via conformational changes and hydrogen-bonded connections.

## 5. Conclusions

The hydration level of CcO depends on the position of the excess proton. A proton on K362 or at the height of K362 leads to a high number of water molecules in the channel. With high hydration, probabilities to form hydrogen bonds between protein residues and water molecules and thus also hydrogen-bonded chains are significantly higher than in “dry” models. In particular, proton passage of S365 is hindered by the low water density between this residue and K362 when the excess proton is located below S365. The hydration level at the channel entrance, however, is sufficient to allow proton transport from H96 as initial proton acceptor, supported by nearby E101, up to the height of S365. Once the excess proton has passed S365, which appears to be the rate-determining step, channel hydration is sufficient to allow proton transport by a hydronium ion and its moving water cloud up to K362 and beyond. The water cloud not only moves but also drags further water inside the channel, leading to a widening of the channel such that a conformational change of K362 is facilitated. This conformational change takes place partially when the hydronium ion is close to K362, i.e., before proton transfer to K362, and is completed with protonated K362. The high number of water molecules, hydrogen-bond lifetimes of 10 ps or more and the resulting hydrogen-bonded connectivity, render proton transfer from protonated K362 to Y288 via hydrogen-bonded chains highly feasible.

## Figures and Tables

**Table 1:** Protonation models of CcO.

Model	Protonated K-Channel Residues
0100	-
0101	E101
0100H	H96
0110	K362
0111	K362 and E101
0110H	K362 and H96
0100a	H${}_{3}$O${}^{+}$ in position a
0100b	H${}_{3}$O${}^{+}$ in position b
0100c	H${}_{3}$O${}^{+}$ in position c
0100d	H${}_{3}$O${}^{+}$ in position d
0100e	H${}_{3}$O${}^{+}$ in position e
0100f	H${}_{3}$O${}^{+}$ in position f

**Table 2:** Average number of water molecules in the K-channel and volume of the polyhedron (in nm${}^{3}$) used to define the K-channel of CcO, modelled in different protonation states.

Model	Water in Cylinder	Water in Polyhedron	Polyhedron Volume [nm${}^{3}$]
0100f	13.7 ± 3.3	6.6 ± 1.3	3.270 ± 0.036
0100e	16.7 ± 3.6	10.0 ± 2.6	3.259 ± 0.043
0100d	24.0 ± 2.5	17.2 ± 1.3	3.336 ± 0.016
0110H	31.2 ± 9.1	18.2 ± 3.1	3.429 ± 0.120
0111	15.5 ± 0.7	12.1 ± 0.5	3.266 ± 0.057
0110	30.1 ± 4.2	19.4 ± 0.9	3.422 ± 0.050
0100c	19.9 ± 2.2	11.8 ± 1.6	3.278 ± 0.020
0100b	9.5 ± 2.4	5.4 ± 1.1	3.149 ± 0.046
0100a	9.9 ± 4.2	7.3 ± 1.1	3.198 ± 0.019
0100H	12.0 ± 0.3	6.5 ± 0.5	3.186 ± 0.026
0101	6.7 ± 2.0	5.5 ± 4.1	3.122 ± 0.008
0100	7.5 ± 4.4	2.7 ± 3.4	3.182 ± 0.017

**Table 3:** Average distances (in Å) between important residues in the K-channel of CcO, modelled in different protonation states. For further distances and the probability distributions of distances, see Supplementary Information.

Model	H96-Y288	E101-Y288	K362-Y288	P358-A319	M316-K365	P315-E101	P315-S365
0100f	26.9 ± 2.6	21.5 ± 0.1	8.3 ± 0.1	5.4 ± 0.0	6.3 ± 0.1	5.6 ± 0.3	9.3 ± 0.2
0100e	26.8 ± 1.9	23.1 ± 0.3	10.7 ± 0.4	5.5 ± 0.1	6.8 ± 0.2	6.0 ± 0.3	9.4 ± 0.1
0100d	25.9 ± 0.5	22.2 ± 0.5	12.6 ± 2.2	6.3 ± 0.3	7.4 ± 0.2	5.9 ± 0.0	10.0 ± 0.0
0110H	26.8 ± 2.7	22.3 ± 0.4	10.8 ± 0.2	7.2 ± 0.5	7.9 ± 0.4	6.7 ± 0.5	10.2 ± 0.4
0111	30.6 ± 2.6	21.3 ± 0.7	11.0 ± 0.5	6.4 ± 0.6	7.5 ± 0.2	5.5 ± 0.5	9.9 ± 0.3
0110	28.0 ± 4.6	21.9 ± 0.3	10.9 ± 0.1	7.2 ± 0.3	7.9 ± 0.3	6.3 ± 0.2	10.4 ± 0.3
0100c	26.3 ± 0.7	21.7 ± 0.2	10.0 ± 0.1	6.5 ± 0.7	7.6 ± 0.2	6.3 ± 0.2	9.6 ± 0.2
0100b	26.5 ± 1.0	22.0 ± 0.9	13.0 ± 0.3	5.3 ± 0.1	6.7 ± 0.1	5.4 ± 0.6	9.2 ± 0.0
0100a	29.7 ± 0.3	23.2 ± 0.0	13.2 ± 0.2	5.3 ± 0.1	6.6 ± 0.0	6.3 ± 0.1	9.3 ± 0.0
0100H	25.4 ± 1.0	23.4 ± 0.1	13.0 ± 0.3	5.4 ± 0.2	6.7 ± 0.2	6.2 ± 0.3	9.4 ± 0.1
0101	28.0 ± 1.7	21.3 ± 0.9	12.4 ± 0.4	5.2 ± 0.1	6.7 ± 0.1	4.4 ± 0.2	9.2 ± 0.2
0100	29.0 ± 1.1	23.4 ± 0.1	12.8 ± 0.7	5.3 ± 0.1	6.6 ± 0.1	5.8 ± 0.3	9.3 ± 0.1

**Table 4:** Average number of hydrogen bonds between important protein residues and water in the K-channel of CcO, modelled in different protonation states.

Model	Y288	T359	K362	S365	H96	E101	H${}_{3}$O+
0100f	0.7 ± 0.5	0.8 ± 0.4	0.9 ± 0.1	0.8 ± 0.0	0.4 ± 0.1	4.0 ± 0.3	1.3 ± 0.4
0100e	1.0 ± 0.3	1.5 ± 0.1	0.4 ± 0.1	1.1 ± 0.1	0.2 ± 0.0	3.4 ± 0.2	1.1 ± 0.2
0100d	1.1 ± 0.1	1.6 ± 0.0	0.4 ± 0.2	0.9 ± 0.0	0.3 ± 0.1	3.8 ± 0.6	2.2 ± 0.4
0110H	1.2 ± 0.1	1.6 ± 0.1	2.5 ± 0.0	1.2 ± 0.0	0.4 ± 0.1	3.8 ± 0.6	-
0111	1.0 ± 0.0	1.5 ± 0.1	2.6 ± 0.1	0.9 ± 0.1	0.2 ± 0.1	2.5 ± 1.7	-
0110	1.0 ± 0.1	1.6 ± 0.2	2.5 ± 0.1	1.1 ± 0.1	0.4 ± 0.0	4.1 ± 0.6	-
0100c	0.7 ± 0.2	1.2 ± 0.0	0.8 ± 0.0	0.6 ± 0.4	0.1 ± 0.1	1.5 ± 0.4	2.6 ± 0.2
0100b	0.9 ± 0.0	1.1 ± 0.0	0.7 ± 0.2	0.0 ± 0.0	0.1 ± 0.1	2.7 ± 0.9	1.1 ± 0.4
0100a	0.3 ± 0.1	1.1 ± 0.1	0.9 ± 0.1	0.9 ± 0.0	0.1 ± 0.1	1.9 ± 0.3	0.6 ± 0.8
0100H	0.9 ± 0.0	1.0 ± 0.1	0.9 ± 0.0	0.8 ± 0.0	0.1 ± 0.0	2.4 ± 0.1	-
0101	0.5 ± 0.4	1.0 ± 0.2	0.9 ± 0.1	0.7 ± 0.1	0.1 ± 0.1	1.0 ± 0.7	-
0100	0.5 ± 0.4	1.1 ± 0.2	0.8 ± 0.1	0.3 ± 0.4	0.1 ± 0.1	1.1 ± 1.4	-

**Table 5:** Lifetimes (in ps) of hydrogen bonds between important K-channel residues and water. “N/A” marks life times for which the error is larger than the calculated average value.

Model	Y288	T359	K362	S365	H96	E101
0100f	<2	4.3 ± 3.7	15.3 ± 1.8	33.5 ± 22.3	7.6 ± 1.9	N/A
0100e	23.7 ± 14.0	92.1 ± 24.6	16.8 ± 8.9	62.4 ± 28.1	6.6 ± 2.1	1078.9 ± 353.9
0100d	39.8 ± 5.8	109.9 ± 16.5	N/A	41.3 ± 36.0	6.9 ± 1.0	1293.7 ± 356.3
0110H	38.2 ± 6.1	121.3 ± 47.0	N/A	46.8 ± 12.7	21.3 ± 3.6	N/A
0111	21.5 ± 6.8	N/A	1896.5 ± 713.3	16.1 ± 5.6	7.1 ± 1.2	N/A
0110	26.7 ± 7.4	93.1 ± 25.3	1373.0 ± 139.6	28.5 ± 4.7	10.7 ± 3.6	N/A
0100c	297.9 ± 173.7	N/A	18.7 ± 4.1	17.9 ± 6.8	6.8 ± 1.4	N/A
0100b	12.4 ± 0.4	33.4 ± 4.6	10.6 ± 1.0	N/A	6.6 ± 1.1	5999.4 ± 4023.1
0100a	12.2 ± 0.2	39.2 ± 8.9	14.7 ± 2.9	<2	5.0 ± 1.0	110.8 ± 77.9
0100H	12.6 ± 0.6	35.6 ± 1.9	18.2 ± 5.0	15.5 ± 1.7	24.9 ± 12.7	964.1 ± 642.6
0101	9.1 ± 4.8	38.6 ± 5.6	27.9 ± 12.4	13.7 ± 2.3	<2	135.9 ± 102.6
0100	10.4 ± 4.9	53.2 ± 31.1	22.3 ± 8.3	21.8 ± 4.8	3.2 ± 2.3	676.3 ± 459.8

## Data Availability

The data presented in this study are available on request from the corresponding author.

## Supplementary Materials

The following supporting information can be downloaded at: https://www.mdpi.com/article/10.3390/biom12111615/s1, Table S1: Average accumulated number of water molecules along the cylinder axis; Table S2: Average number of water molecules in the K-channel of CcO, at different distances to the cylinder axis; Table S3: Average distances (in Å) between important residues in the K-channel of CcO, modelled in different protonation states; Figure S1: Median structures of the K-channel of CcO from the MD simulations with different protonation states; Figure S2: Water mobility in the different components; Figure S3: Projection of water occupancy in the K-channel of CcO with different protonation states; Figures S4–S14: Probability distributions of side chain dihedral angles of protein residues; Figures S15–S25: Probability distributions of distances between protein residues.

## Abbreviations

The following abbreviations are used in this manuscript:

CcO	Cytochrome c Oxidase
MD	Molecular dynamics
BNC	bi-nuclear centre

## References

[1] Wikstrom M.K. (1977). Proton pump coupled to cytochrome c oxidase in mitochondria. Nature.

[2] Iwata S., Ostermeier C., Ludwig B., Michel H. (1995). Structure at 2.8 Å resolution of cytochrome c oxidase from Paracoccus denitrificans. Nature.

[3] Konstantinov A.A., Siletsky S., Mitchell D., Kaulen A., Gennis R.B. (1997). The roles of the two proton input channels in cytochrome c oxidase from Rhodobacter sphaeroides probed by the effects of site-directed mutations on time-resolved electrogenic intraprotein proton transfer. Proc. Nat. Acad. Sci. USA.

[4] Belevich I., Bloch D.A., Belevich N., Wikström M., Verkhovsky M.I. (2007). Exploring the proton pump mechanism of cytochrome c oxidase in real time. Proc. Nat. Acad. Sci. USA.

[5] Qin L., Hiser C., Mulichak A., Garavito R.M., Ferguson-Miller S. (2006). Identification of conserved lipid/detergent-binding sites in a high-resolution structure of the membrane protein cytochrome c oxidase. Proc. Nat. Acad. Sci. USA.

[6] Ghane T., Gorriz R.F., Wrzalek S., Volkenandt S., Dalatieh F., Reidelbach M., Imhof P. (2018). Hydrogen-Bonded Network and Water Dynamics in the D-channel of Cytochrome c Oxidase. J. Membr. Biol..

[7] Reidelbach M., Imhof P. (2020). Proton transfer in the D-channel of cytochrome c oxidase modeled by a transition network approach. Biochim. Biophys. Acta Gen. Subj..

[8] Lee S., Liang R., Voth G.A., Swanson J.M.J. (2016). Computationally Efficient Multiscale Reactive Molecular Dynamics to Describe Amino Acid Deprotonation in Proteins. J. Chem. Theory Comput..

[9] Woelke A.L., Galstyan G., Knapp E.W. (2014). Lysine 362 in cytochrome c oxidase regulates opening of the K-channel via changes in pKA and conformation. Biochim. Biophys. Acta (BBA) Bioenerg..

[10] Vygodina T., Pecoraro C., Mitchell D., Gennis R., Konstantinov A. (1998). Mechanism of inhibition of electron transfer by amino acid replacement K362M in a proton channel of Rhodobacter sphaeroides cytochrome c oxidase. Biochemistry.

[11] Brzezinski P., Adelroth P. (1998). Proton-controlled electron transfer in cytochrome c oxidase: Functional role of the pathways through Glu 286 and Lys 362. Acta Physiol. Scand. Suppl..

[12] Tuukkanen A., Verkhovsky M.I., Laakkonen L., Wikström M. (2006). The K-pathway revisited: A computational study on cytochrome c oxidase. Biochim. Biophys. Acta (BBA) Bioenerg..

[13] Supekar S., Kaila V.R.I. (2018). Dewetting transitions coupled to K-channel activation in cytochrome c oxidase. Chem. Sci..

[14] Stegmaier V., Gorriz R.F., Imhof P. (2021). Protonation Dynamics in the K-Channel of Cytochrome c Oxidase Estimated from Molecular Dynamics Simulations. Processes.

[15] Wolf A., Dragelj J., Wonneberg J., Stellmacher J., Balke J., Woelke A.L., Hodoscek M., Knapp E.W., Alexiev U. (2020). The redox-coupled proton-channel opening in cytochrome c oxidase. Chem. Sci..

[16] Peng Y., Swanson J.M., Kang S.G., Zhou R., Voth G.A. (2014). Hydrated excess protons can create their own water wires. J. Phys. Chem. B.

[17] Farahvash A., Stuchebrukhov A. (2018). Investigating the Many Roles of Internal Water in Cytochrome c Oxidase. J. Phys. Chem. B.

[18] Olkhova E., Hutter M.C., Lill M.A., Helms V., Michel H. (2004). Dynamic Water Networks in Cytochrome c Oxidase from Paracoccus denitrificans Investigated by Molecular Dynamics Simulations. Biophys. J..

[19] Son C.Y., Yethiraj A., Cui Q. (2017). Cavity hydration dynamics in cytochrome c oxidase and functional implications. Proc. Nat. Acad. Sci. USA.

[20] Cukier R. (2005). A molecular dynamics study of water chain formation in the proton-conducting K channel of cytochrome c oxidase. Biochim. Et Biophys. Acta (BBA)—Bioenerg..

[21] Yang L., Skjevik. Å.A., Han Du W.G., Noodleman L., Walker R.C., Götz A.W. (2016). Water exit pathways and proton pumping mechanism in B-type cytochrome c oxidase from molecular dynamics simulations. Biochim. Et Biophys. Acta (BBA) Bioenerg..

[22] Helabad M.B., Ghane T., Reidelbach M., Woelke A.L., Knapp E.W., Imhof P. (2017). Protonation-State-Dependent Communication in Cytochrome c Oxidase. Biophys. J..

[23] Sagnella D., Voth G. (1996). Structure and dynamics of hydronium in the ion channel gramicidin A. Biophys. J..

[24] Jorgensen W.L., Chandrasekhar J., Madura J.D., Impey R.W., Klein M.L. (1983). Comparison of simple potential functions for simulating liquid water. J. Chem. Phys..

[25] MacKerell A.D., Bashford D., Bellott M., Dunbrack R.L., Evanseck J.D., Field M.J., Fischer S., Gao J., Guo H., Ha S. (1998). All-Atom Empirical Potential for Molecular Modeling and Dynamics Studies of Proteins. J. Phys. Chem. B..

[26] Klauda J.B., Venable R.M., Freites J.A., O’Connor J.W., Tobias D.J., Mondragon-Ramirez C., Vorobyov I., MacKerell A.D., Pastor R.W. (2010). Update of the CHARMM all-atom additive force field for lipids: Validation on six lipid types. J. Phys. Chem. B.

[27] Darden T., York D., Pedersen L.G. (1993). Particle mesh Ewald: An Nlog(N) method for Ewald sums in large systems. J. Chem. Phys..

[28] Ryckaert J.P., Ciccotti G., Berendsen H.J.C. (1977). Numerical integration of the cartesian equations of motion of a system with constraints: Molecular dynamics of n-alkanes. J. Comp. Phys..

[29] Phillips J.C., Braun R., Wang W., Gumbart J., Tajkhorshid E., Villa E., Chipot C., Skeel R.D., Kale L., Schulten K. (2005). Scalable molecular dynamics with NAMD. J. Comput. Chem..

[30] Gowers R.J., Linke M., Barnoud J., Reddy T.J.E., Melo M.N., Seyler S.L., Domanski J., Dotson D.L., Buchoux S., Kenney I.M. (2019). MDAnalysis: A Python Package for the Rapid Analysis of Molecular Dynamics Simulations.

[31] Bagga J., Heinz A., Mutzel P., Jünger M., Leipert S. (2002). JGraph—A Java Based System for Drawing Graphs and Running Graph Algorithms. Graph Drawing.

[32] Humphrey W., Dalke A., Schulten K. (1996). VMD—Visual Molecular Dynamics. J. Mol. Graph..

[33] Hunter J.D. (2007). Matplotlib: A 2D graphics environment. Comput. Sci. Eng..

[34] Dijkstra E. (1959). A note on two problems in connexion with graphs. Num. Math..

[35] Brändén M., Tomson F., Gennis R.B., Brzezinski P. (2002). The entry point of the K-proton-transfer pathway in cytochrome c oxidase. Biochemistry.

[36] Hiser C., Liu J., Ferguson-Miller S. (2018). The K-path entrance in cytochrome c oxidase is defined by mutation of E101 and controlled by an adjacent ligand binding domain. Biochim. Biophys. Acta Bioenerg..

[37] Brändén M., Sigurdson H., Namslauer A., Gennis R.B., Ädelroth P., Brzezinski P. (2001). On the role of the K-proton transfer pathway in cytochrome c oxidase. Proc. Nat. Acad. Sci. USA.

[38] Cai X., Haider K., Lu J., Radic S., Son C.Y., Cui Q., Gunner M. (2018). Network analysis of a proposed exit pathway for protons to the P-side of cytochrome c oxidase. Biochim. Et Biophys. Acta (BBA) Bioenerg..

