# Supplementary material for: Interplay of Hydration and Protonation Dynamics in the K-Channel of Cytochrome c Oxidase
Source: Biomolecules. 2022 Nov 1;12(11):1615. doi: 10.3390/biom12111615 (PMC9687966; doi:10.3390/biom12111615)
Supplement: Supplementary file 1 [file biomolecules-12-01615-s001.zip › biomolecules-1962882-supplementary.pdf]

Supplementary Information for Interplay of Hydration, Water Mobility, and Proton Transfer in the K-channel of CcO

Rene F. Gorriz and Petra Imhof

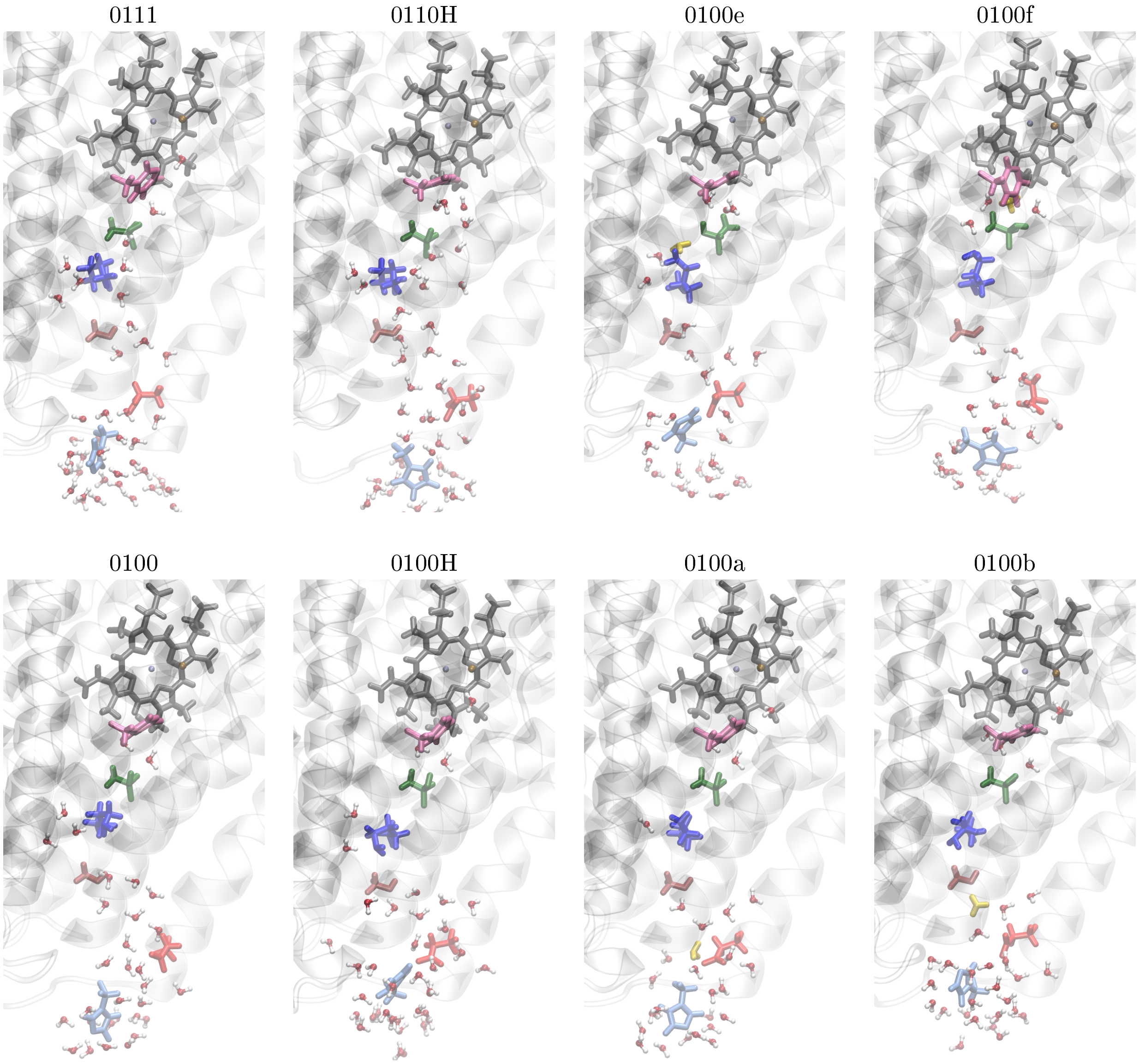

Figure S1: Median structures of the K-channel of CcO from the MD simulations with different protonation states. Important protein residues are marked by their labels and colours: H96 (light blue), E101 (red), S365 (brown), K362 (blue), T359 (dark green), Y288 (magenta), and the H_3O^+ ion (yellow), if present.

Water

Table S1: Average accumulated number of water molecules along the cylinder axis in the K-Channel of CcO.

Height		0110	0111	0110H	0100d	0100e	0100f
$\leq$ 13.0		30.1 $\pm$ 4.2	15.5 $\pm$ 0.7	30.9 $\pm$ 8.6	24.0 $\pm$ 2.5	16.7 $\pm$ 3.6	13.7 $\pm$ 3.3
$\leq$ 11.0	▼	29.4 $\pm$ 3.2	15.3 $\pm$ 0.9	30.3 $\pm$ 8.1	23.7 $\pm$ 2.4	16.7 $\pm$ 3.6	13.7 $\pm$ 3.3
$\leq$ 9.0		27.8 $\pm$ 3.2	14.2 $\pm$ 0.4	28.8 $\pm$ 7.7	22.7 $\pm$ 1.9	15.8 $\pm$ 3.5	13.7 $\pm$ 3.3
$\leq$ 7.0		26.3 $\pm$ 2.4	13.5 $\pm$ 0.2	27.3 $\pm$ 6.8	21.4 $\pm$ 1.6	15.7 $\pm$ 3.4	13.0 $\pm$ 3.4
$\leq$ 5.0		23.7 $\pm$ 2.3	11.4 $\pm$ 0.7	25.4 $\pm$ 6.7	18.9 $\pm$ 1.5	13.9 $\pm$ 3.0	11.9 $\pm$ 3.3
$\leq$ 3.0	◀	21.6 $\pm$ 2.2	9.7 $\pm$ 0.9	23.7 $\pm$ 6.7	16.8 $\pm$ 1.6	13.5 $\pm$ 2.5	10.9 $\pm$ 3.2
$\leq$ 1.0		19.5 $\pm$ 2.8	7.7 $\pm$ 1.0	21.4 $\pm$ 6.8	14.3 $\pm$ 1.2	12.5 $\pm$ 1.7	10.7 $\pm$ 3.2
$\leq$ -1.0	✖	17.8 $\pm$ 2.8	6.6 $\pm$ 0.9	19.8 $\pm$ 6.6	12.8 $\pm$ 0.9	10.8 $\pm$ 2.0	10.7 $\pm$ 3.2
$\leq$ -3.0		16.1 $\pm$ 2.9	5.4 $\pm$ 0.8	17.9 $\pm$ 6.4	11.2 $\pm$ 0.8	9.8 $\pm$ 2.2	10.7 $\pm$ 3.2
$\leq$ -5.0		14.4 $\pm$ 2.7	4.7 $\pm$ 0.5	15.8 $\pm$ 6.2	10.0 $\pm$ 1.1	9.4 $\pm$ 1.8	10.5 $\pm$ 3.3
$\leq$ -7.0	▶	12.2 $\pm$ 2.4	3.8 $\pm$ 0.2	13.3 $\pm$ 5.8	8.7 $\pm$ 1.0	7.7 $\pm$ 1.0	10.0 $\pm$ 3.4
$\leq$ -9.0		9.3 $\pm$ 2.3	2.5 $\pm$ 0.3	10.2 $\pm$ 5.6	6.5 $\pm$ 0.8	5.6 $\pm$ 0.5	8.1 $\pm$ 3.6
$\leq$ -11.0		6.5 $\pm$ 2.2	2.2 $\pm$ 0.3	6.9 $\pm$ 4.5	4.3 $\pm$ 0.6	3.8 $\pm$ 0.6	6.1 $\pm$ 3.2
$\leq$ -13.0	■	3.7 $\pm$ 1.3	1.2 $\pm$ 0.5	4.1 $\pm$ 3.2	2.3 $\pm$ 0.6	2.0 $\pm$ 0.6	3.4 $\pm$ 2.4
$\leq$ -15.0	▲	1.4 $\pm$ 0.5	0.2 $\pm$ 0.0	1.5 $\pm$ 1.2	0.7 $\pm$ 0.2	0.7 $\pm$ 0.1	1.4 $\pm$ 1.1

Height		0100	0101	0100H	0100a	0100b	0100c
$\leq$ 13.0		7.5 $\pm$ 4.4	6.7 $\pm$ 2.0	12.0 $\pm$ 0.3	9.9 $\pm$ 4.2	9.5 $\pm$ 2.4	19.9 $\pm$ 2.2
$\leq$ 11.0	▼	7.2 $\pm$ 4.5	6.7 $\pm$ 2.0	11.8 $\pm$ 0.5	9.7 $\pm$ 4.1	9.5 $\pm$ 2.4	19.2 $\pm$ 2.1
$\leq$ 9.0		6.9 $\pm$ 4.3	6.1 $\pm$ 1.7	11.0 $\pm$ 0.3	8.4 $\pm$ 4.2	8.4 $\pm$ 2.4	18.4 $\pm$ 2.2
$\leq$ 7.0		6.7 $\pm$ 4.3	6.1 $\pm$ 1.6	10.9 $\pm$ 0.3	8.2 $\pm$ 4.2	8.4 $\pm$ 2.5	17.6 $\pm$ 2.2
$\leq$ 5.0		5.4 $\pm$ 4.0	5.5 $\pm$ 1.5	9.9 $\pm$ 0.5	6.9 $\pm$ 3.8	7.7 $\pm$ 2.3	16.3 $\pm$ 1.7
$\leq$ 3.0	◀	5.0 $\pm$ 4.2	4.8 $\pm$ 1.3	9.3 $\pm$ 0.6	6.4 $\pm$ 3.9	6.7 $\pm$ 2.3	15.6 $\pm$ 1.4
$\leq$ 1.0		4.4 $\pm$ 4.0	3.9 $\pm$ 1.2	8.6 $\pm$ 0.6	5.9 $\pm$ 3.6	6.7 $\pm$ 2.2	15.1 $\pm$ 0.9
$\leq$ -1.0	✖	3.5 $\pm$ 3.8	3.2 $\pm$ 1.0	7.8 $\pm$ 0.7	5.4 $\pm$ 3.3	6.4 $\pm$ 2.2	14.7 $\pm$ 0.5
$\leq$ -3.0		3.2 $\pm$ 3.8	2.2 $\pm$ 0.9	7.4 $\pm$ 0.7	4.9 $\pm$ 3.5	5.8 $\pm$ 2.3	14.3 $\pm$ 0.4
$\leq$ -5.0		3.1 $\pm$ 3.8	2.1 $\pm$ 0.8	7.2 $\pm$ 0.6	4.8 $\pm$ 3.4	5.7 $\pm$ 2.4	11.9 $\pm$ 0.6
$\leq$ -7.0	▶	3.0 $\pm$ 3.6	2.1 $\pm$ 0.8	7.0 $\pm$ 0.6	4.6 $\pm$ 3.2	5.3 $\pm$ 2.2	9.9 $\pm$ 1.3
$\leq$ -9.0		2.3 $\pm$ 2.8	1.6 $\pm$ 1.0	5.4 $\pm$ 0.9	3.3 $\pm$ 2.4	4.5 $\pm$ 1.7	7.2 $\pm$ 1.3
$\leq$ -11.0		1.6 $\pm$ 2.0	1.1 $\pm$ 0.8	3.2 $\pm$ 0.7	1.6 $\pm$ 1.2	2.9 $\pm$ 1.2	4.6 $\pm$ 1.1
$\leq$ -13.0	■	0.7 $\pm$ 0.9	0.9 $\pm$ 0.7	1.4 $\pm$ 0.5	0.8 $\pm$ 0.6	1.6 $\pm$ 0.7	2.3 $\pm$ 0.9
$\leq$ -15.0	▲	0.2 $\pm$ 0.3	0.1 $\pm$ 0.1	0.5 $\pm$ 0.1	0.3 $\pm$ 0.2	0.4 $\pm$ 0.2	0.7 $\pm$ 0.3

Table S2: Average number of water molecules in the K-channel of CcO, at different distances to the cylinder axis.

Model	r ≤ 1.0	r ≤ 3.5	r ≤ 5.0	r ≤ 6.5	r ≤ 9.5	polyhedron
0100f	0.4 ± 0.0	6.9 ± 1.1	9.7 ± 1.8	12.8 ± 2.2	17.4 ± 3.6	8.5 ± 2.6
0100e	1.2 ± 0.2	8.9 ± 1.2	12.2 ± 1.0	14.4 ± 1.2	17.3 ± 1.1	10.7 ± 1.5
0100d	1.7 ± 0.2	13.4 ± 1.3	21.2 ± 0.7	25.9 ± 0.3	30.7 ± 0.8	20.7 ± 0.7
0110H	1.5 ± 0.2	11.7 ± 1.7	18.0 ± 3.3	23.1 ± 4.9	31.2 ± 9.1	18.2 ± 3.1
0111	1.0 ± 0.1	7.8 ± 0.2	10.5 ± 0.5	12.7 ± 0.7	15.5 ± 0.7	12.1 ± 0.5
0110	1.7 ± 0.5	12.6 ± 0.5	19.7 ± 1.7	24.2 ± 2.3	30.1 ± 4.2	19.4 ± 0.9
0100c	0.4 ± 0.2	5.3 ± 0.2	8.3 ± 0.5	11.1 ± 0.9	15.9 ± 2.4	9.7 ± 4.4
0100b	0.4 ± 0.1	5.8 ± 0.1	8.4 ± 0.1	10.3 ± 0.5	14.0 ± 0.9	11.5 ± 3.9
0100a	0.2 ± 0.2	4.1 ± 1.1	6.5 ± 1.3	8.0 ± 0.9	11.3 ± 1.9	6.9 ± 1.0
0100H	0.3 ± 0.1	4.3 ± 0.4	6.6 ± 0.3	8.6 ± 0.3	12.0 ± 0.3	6.5 ± 0.5
0101	0.1 ± 0.0	2.3 ± 0.5	3.1 ± 0.9	4.2 ± 1.5	6.7 ± 2.0	5.5 ± 4.1
0100	0.2 ± 0.1	2.7 ± 1.7	3.9 ± 2.6	5.0 ± 3.2	7.5 ± 4.4	2.7 ± 3.4

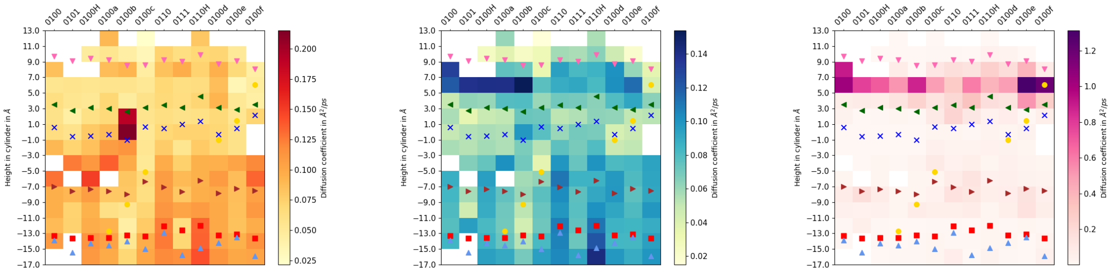

a) b) c)

Figure S2: Water mobility in the different components a) height: along the cylinder Axis, b) radial: distance to cylinder axis , and c) angular component. The symbols mark the average height of protein residues H96 (light blue triangle ▲), E101 (red square ■), S365 (brown triangle-right ▶), K362 (blue cross ✖), T359 (green triangle-left ◀), Y288 (magenta triangle-down ▼), and position of the H_3O^+ ion (yellow circle ●), if present. Residues with an excess proton are marked by a circle around the respective symbol.

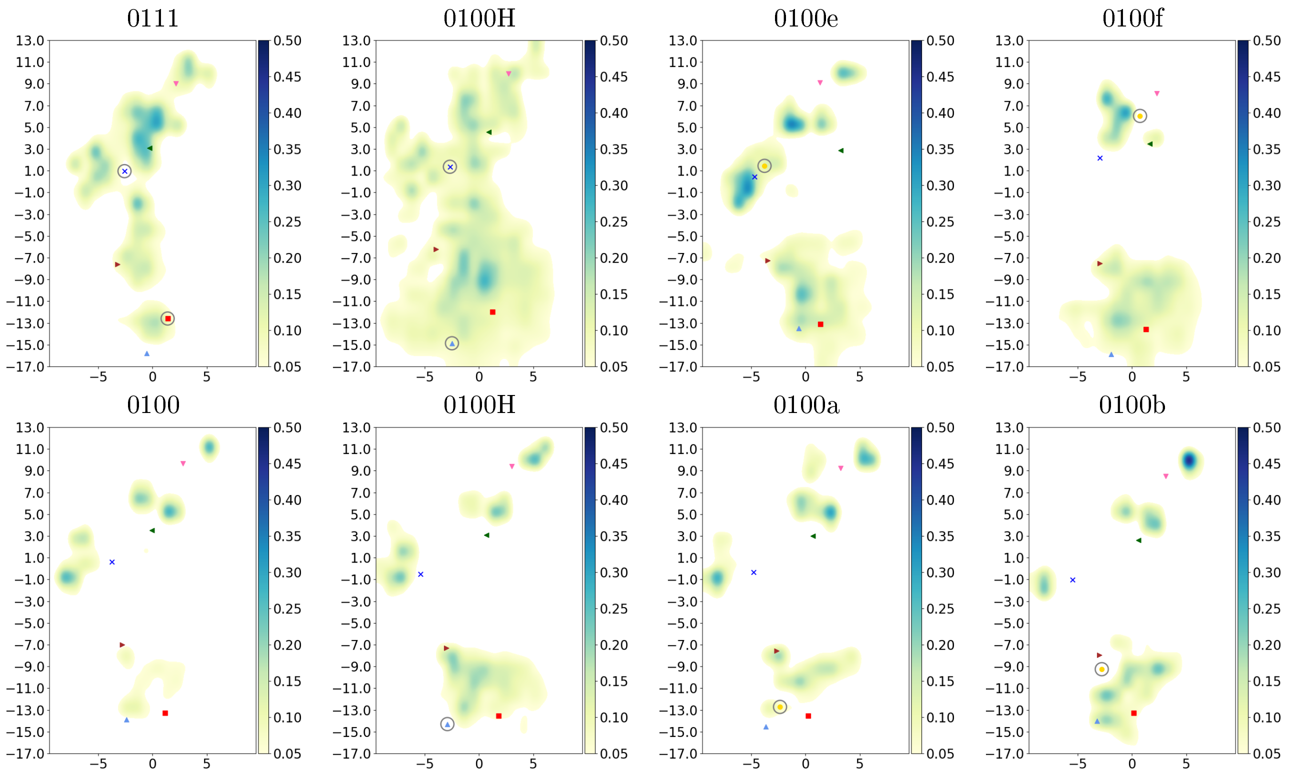

Figure S3: Projection of water occupancy in the K-channel of CcO with different protonation states. The symbols mark the average height of protein residues H96 (light blue triangle ▲), E101 (red square ■), S365 (brown triangle-right ▶), K362 (blue cross ✖), T359 (green triangle-left ◀), Y288 (magenta triangle-down ▼), and position of the H_3O^+ ion (yellow circle ●), if present. Residues with an excess proton are marked by a circle around the respective symbol.

Protein residues

Dihedral angles

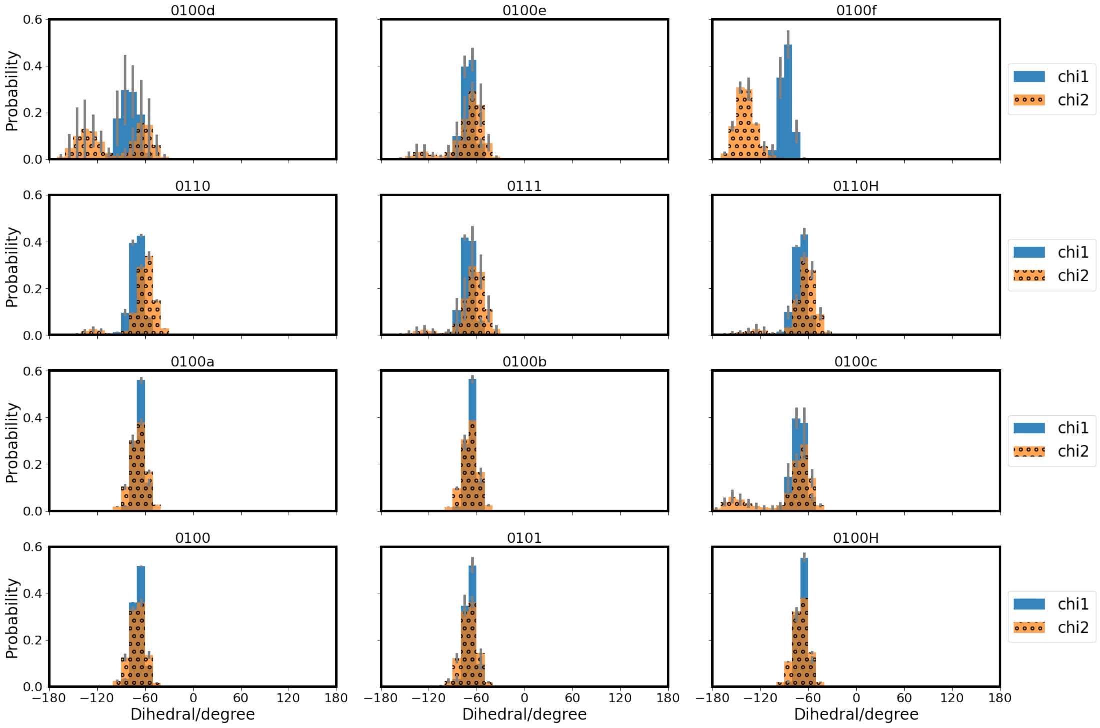

Figure S4: Probability distributions of side chain dihedral angles of Y288.

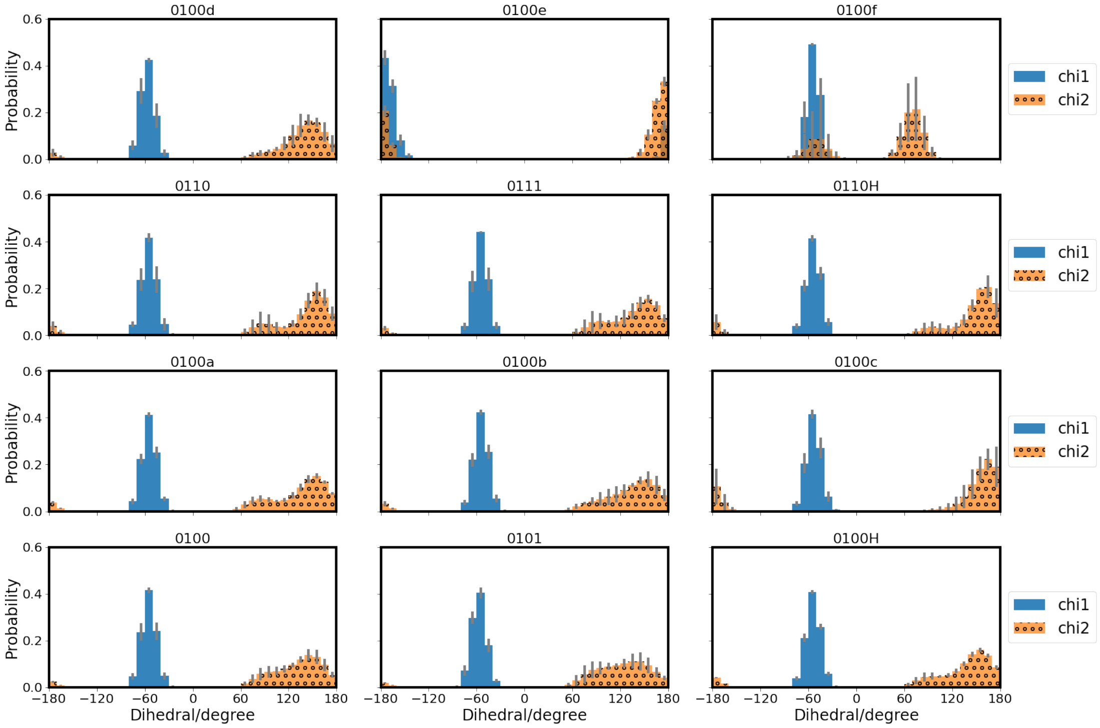

Figure S5: Probability distributions of side chain dihedral angles of T359.

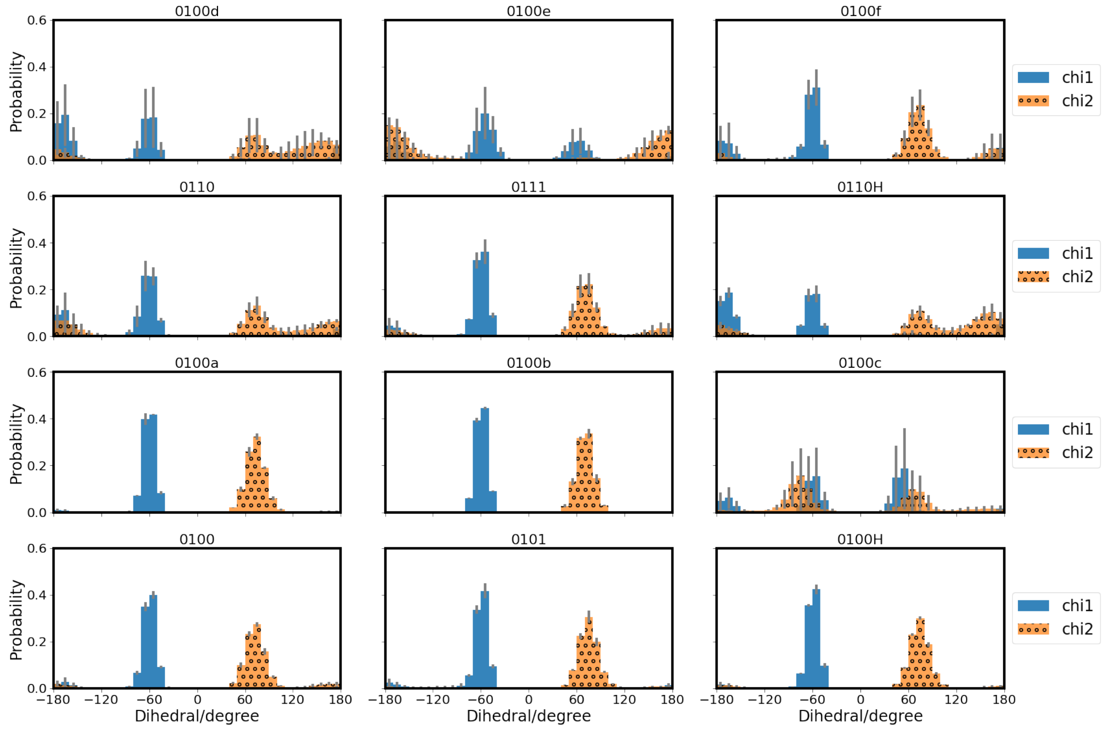

Figure S6: Probability distributions of side chain dihedral angles of S365.

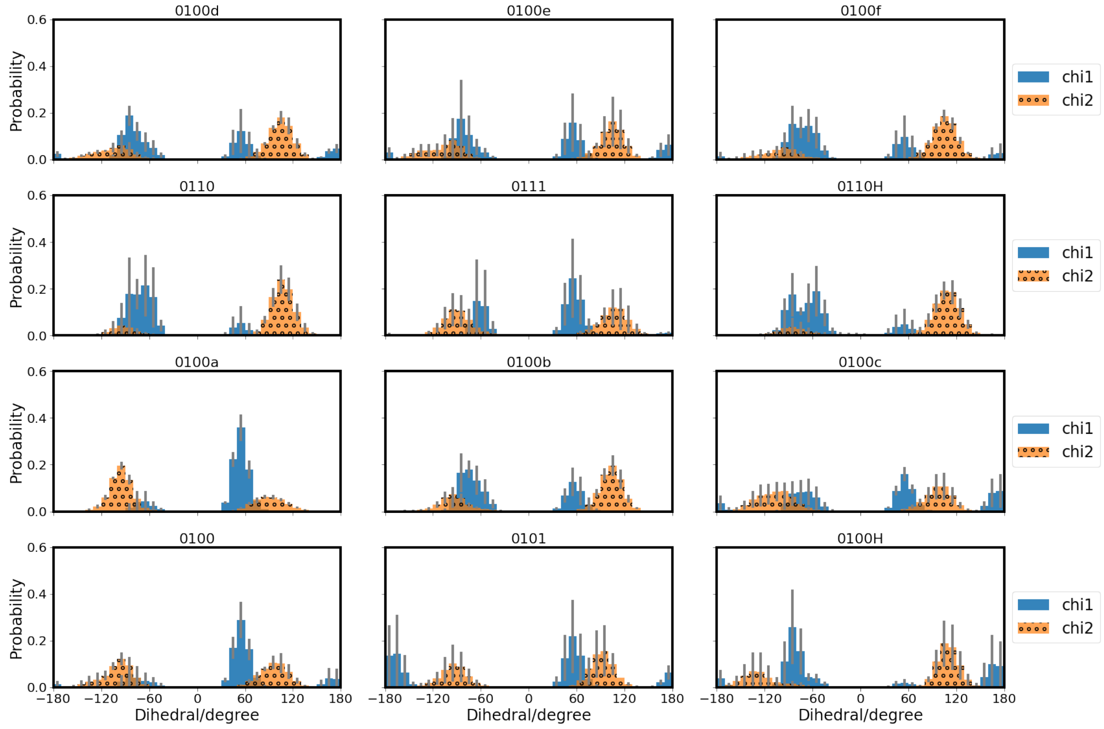

Figure S7: Probability distributions of side chain dihedral angles of H96.

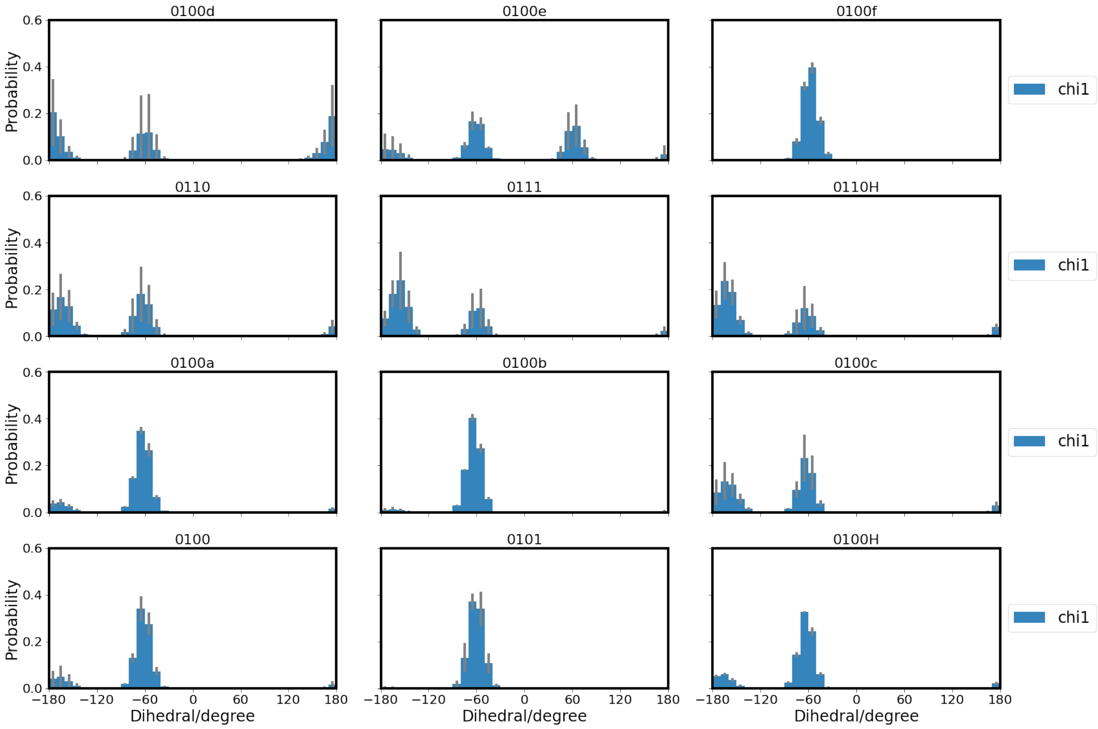

Figure S8: Probability distributions of side chain dihedral angle chi1 of K362.

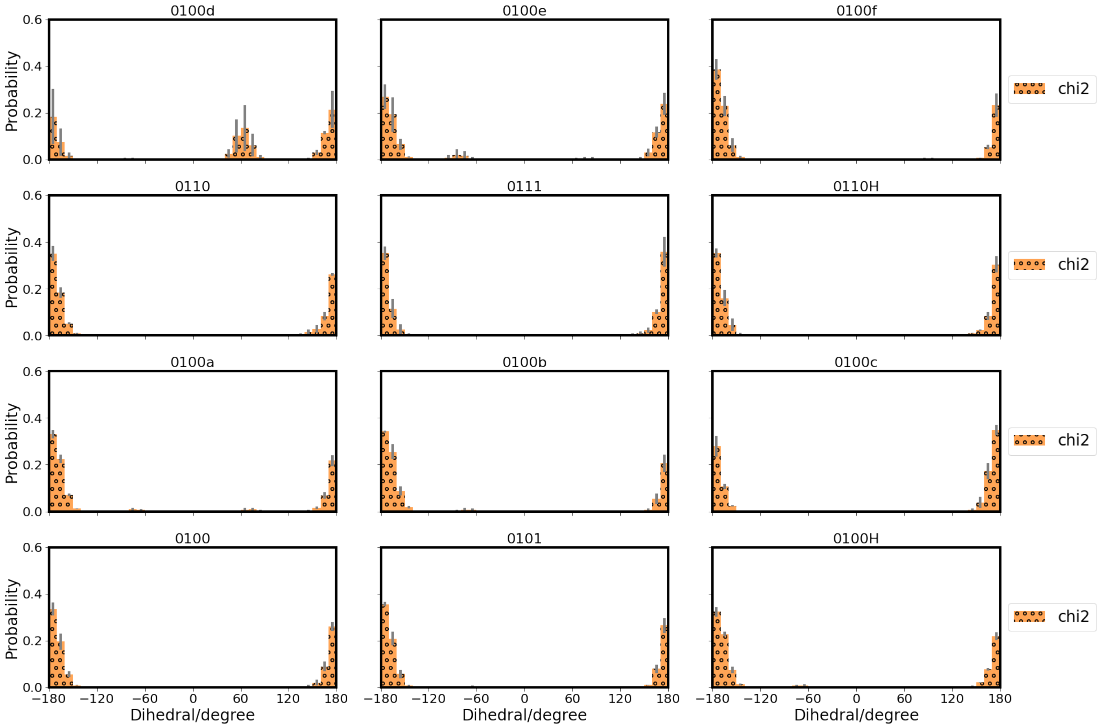

Figure S9: Probability distributions of side chain dihedral angle chi2 of K362.

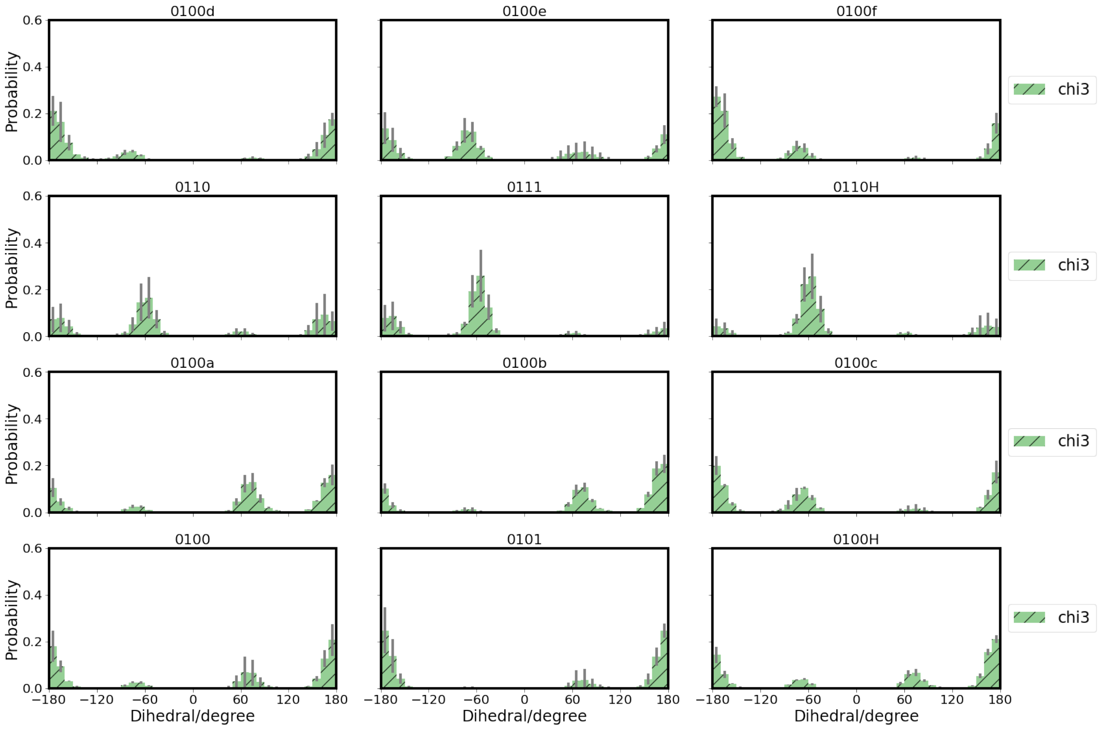

Figure S10: Probability distributions of side chain dihedral angle chi3 of K362.

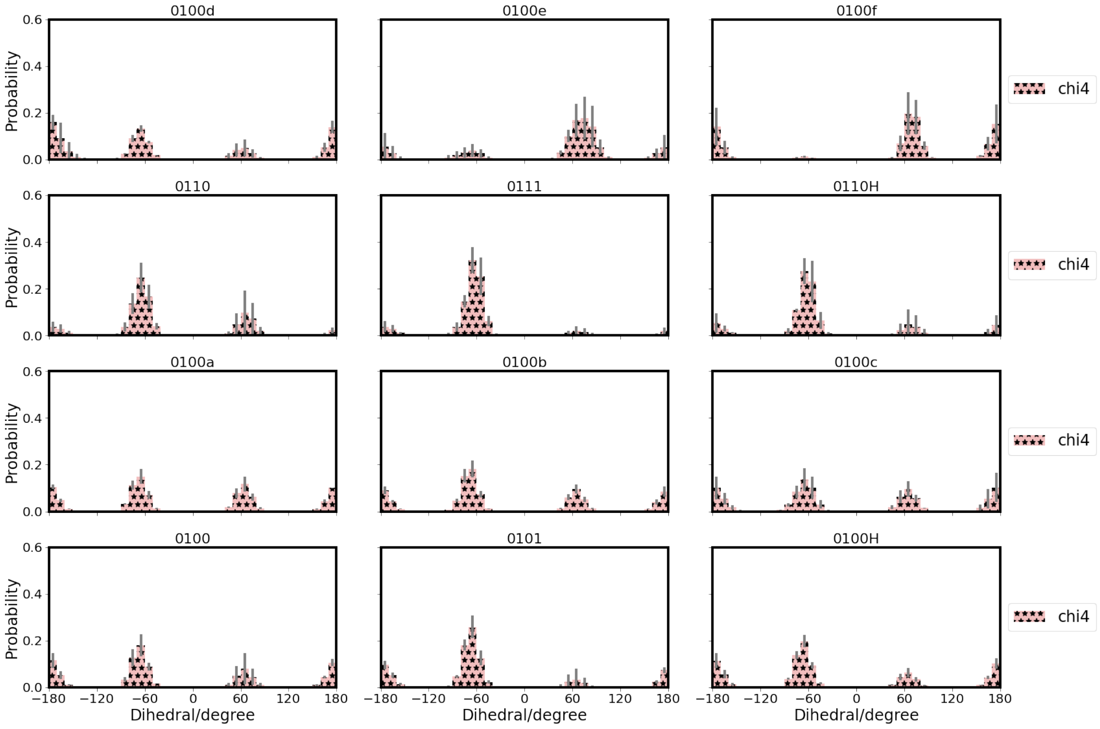

Figure S11: Probability distributions of side chain dihedral angle chi4 of K362.

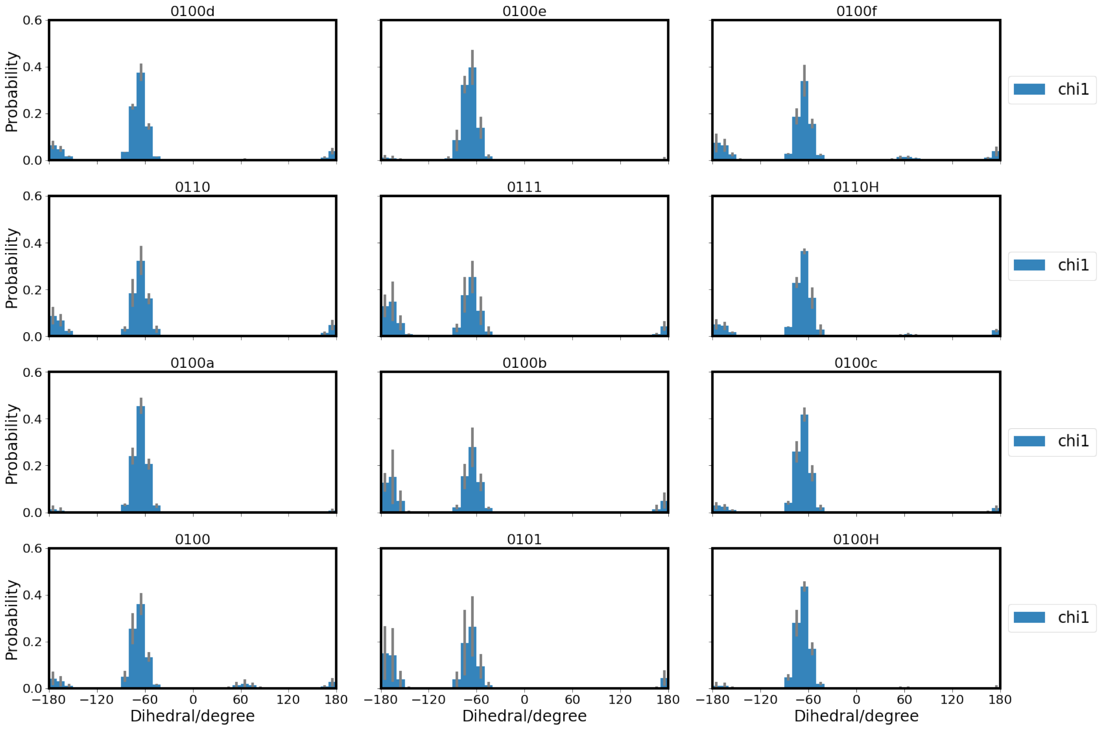

Figure S12: Probability distributions of side chain dihedral angle chi1 of E101.

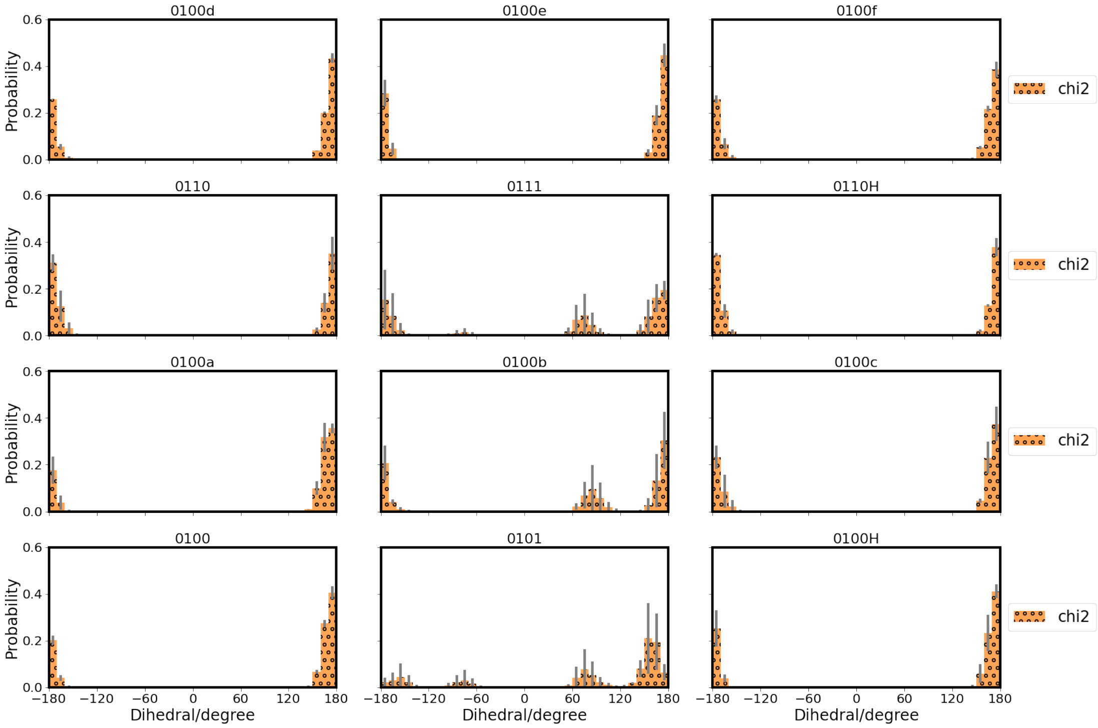

Figure S13: Probability distributions of side chain dihedral angle chi2 of E101.

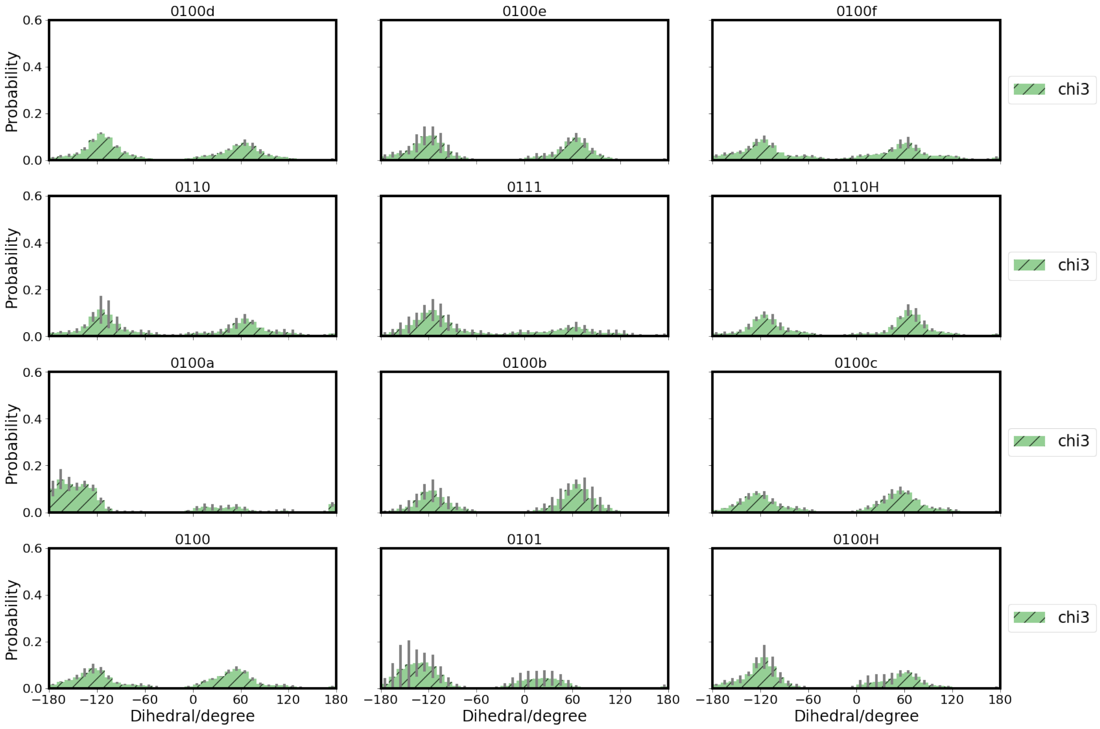

Figure S14: Probability distributions of side chain dihedral angle chi3 of E101.

Distances

Table S3: Average distances (in Å) between important residues in the K-channel of CcO, modelled in different protonation states.

Model	E101-H96	E101-S365	E101-K362	E101-T359	S365-Y288
0100f	7.6 ± 1.8	7.8 ± 0.2	15.1 ± 0.6	17.1 ± 0.7	16.5 ± 0.5
0100e	6.0 ± 1.0	7.7 ± 0.3	15.1 ± 0.3	17.1 ± 0.2	17.6 ± 0.2
0100d	6.1 ± 0.5	7.6 ± 0.3	14.3 ± 0.2	16.6 ± 0.1	17.6 ± 0.3
0110H	6.9 ± 1.6	7.7 ± 0.4	14.1 ± 0.3	16.7 ± 0.3	17.4 ± 0.1
0111	10.3 ± 2.1	6.7 ± 0.4	13.0 ± 0.6	15.5 ± 0.4	17.3 ± 0.4
0110	8.3 ± 3.5	7.2 ± 0.2	13.6 ± 0.2	16.2 ± 0.2	17.5 ± 0.1
0100c	6.4 ± 0.1	8.1 ± 0.2	14.3 ± 0.4	16.5 ± 0.2	16.3 ± 0.4
0100b	7.0 ± 0.9	6.5 ± 0.8	13.1 ± 1.0	15.8 ± 0.8	17.6 ± 0.1
0100a	7.8 ± 0.1	7.5 ± 0.2	14.3 ± 0.1	17.0 ± 0.1	17.8 ± 0.2
0100H	4.6 ± 0.1	7.5 ± 0.0	14.3 ± 0.3	17.1 ± 0.0	17.8 ± 0.1
0101	7.6 ± 2.6	6.0 ± 0.7	12.2 ± 0.7	15.1 ± 1.0	17.8 ± 0.1
0100	7.2 ± 0.7	7.8 ± 0.2	14.4 ± 0.2	17.1 ± 0.0	17.9 ± 0.1

Model	H96-K362	S365-K362	T359-S365	T359-Y288	T359-K362
0100f	19.7 ± 2.8	9.5 ± 0.2	12.0 ± 0.3	4.9 ± 0.6	5.1 ± 1.2
0100e	18.1 ± 1.7	8.4 ± 0.3	10.9 ± 0.1	7.0 ± 0.1	4.5 ± 0.6
0100d	16.2 ± 1.4	8.2 ± 0.9	11.6 ± 0.3	6.1 ± 0.6	7.5 ± 1.5
0110H	17.3 ± 2.6	8.2 ± 0.3	11.7 ± 0.1	5.9 ± 0.1	6.0 ± 0.3
0111	21.3 ± 3.7	8.2 ± 0.4	11.3 ± 0.3	6.2 ± 0.4	5.7 ± 0.1
0110	18.8 ± 5.5	8.4 ± 0.3	11.6 ± 0.1	6.0 ± 0.1	5.7 ± 0.1
0100c	17.7 ± 1.3	8.1 ± 0.2	10.9 ± 0.3	5.5 ± 0.1	5.8 ± 0.6
0100b	15.4 ± 1.0	8.0 ± 0.2	11.0 ± 0.1	6.7 ± 0.1	7.3 ± 0.3
0100a	18.6 ± 0.3	8.0 ± 0.1	11.1 ± 0.1	6.7 ± 0.1	7.5 ± 0.3
0100H	15.0 ± 1.2	8.1 ± 0.2	11.1 ± 0.1	6.7 ± 0.1	7.3 ± 0.4
0101	17.5 ± 1.5	8.1 ± 0.2	11.2 ± 0.2	6.8 ± 0.1	6.8 ± 0.3
0100	18.2 ± 1.4	8.2 ± 0.3	11.1 ± 0.1	6.9 ± 0.1	7.0 ± 0.5

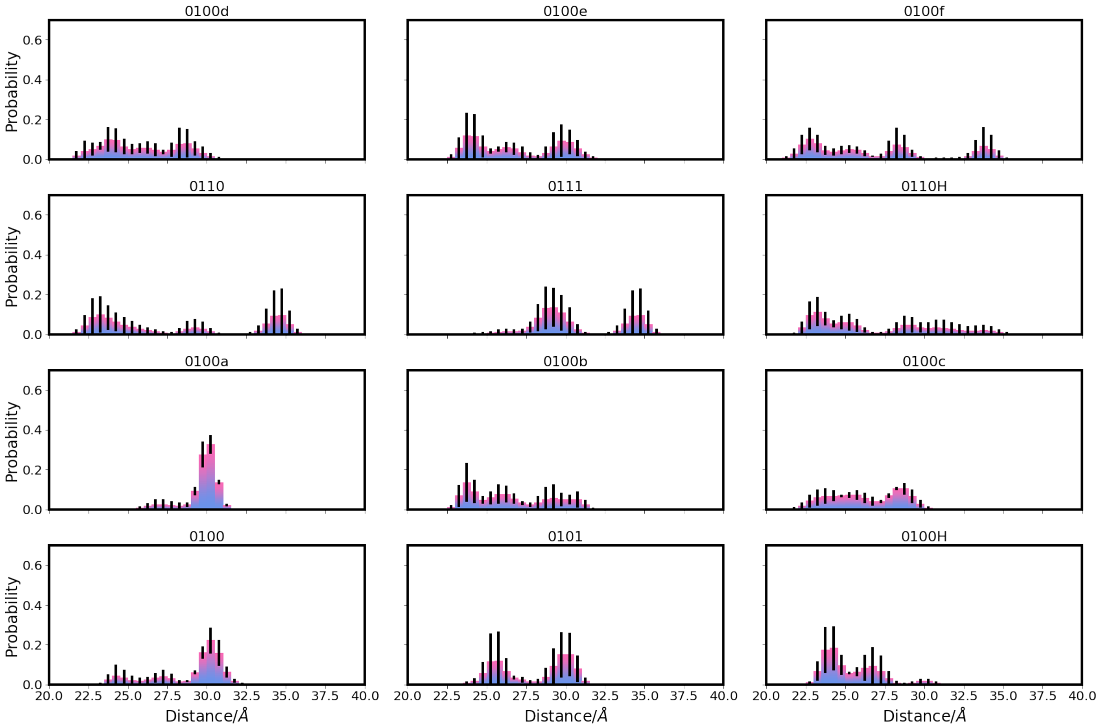

Figure S15: Probability distributions of distances between H96 and Y288.

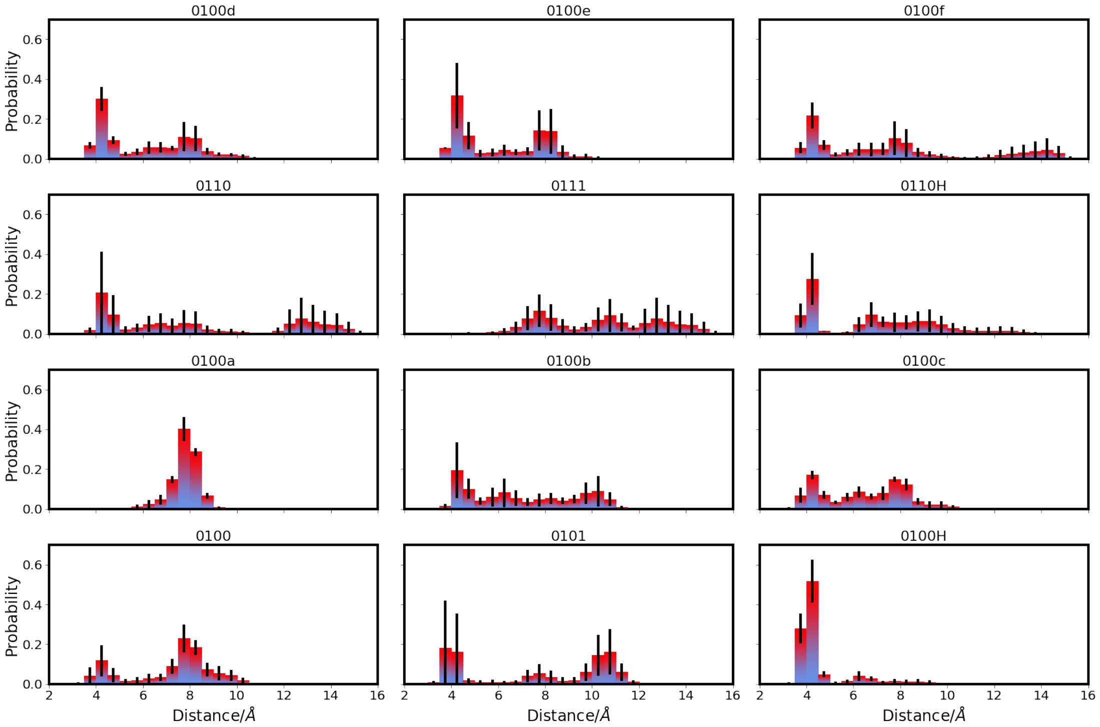

Figure S16: Probability distributions of distances between H96 and E101.

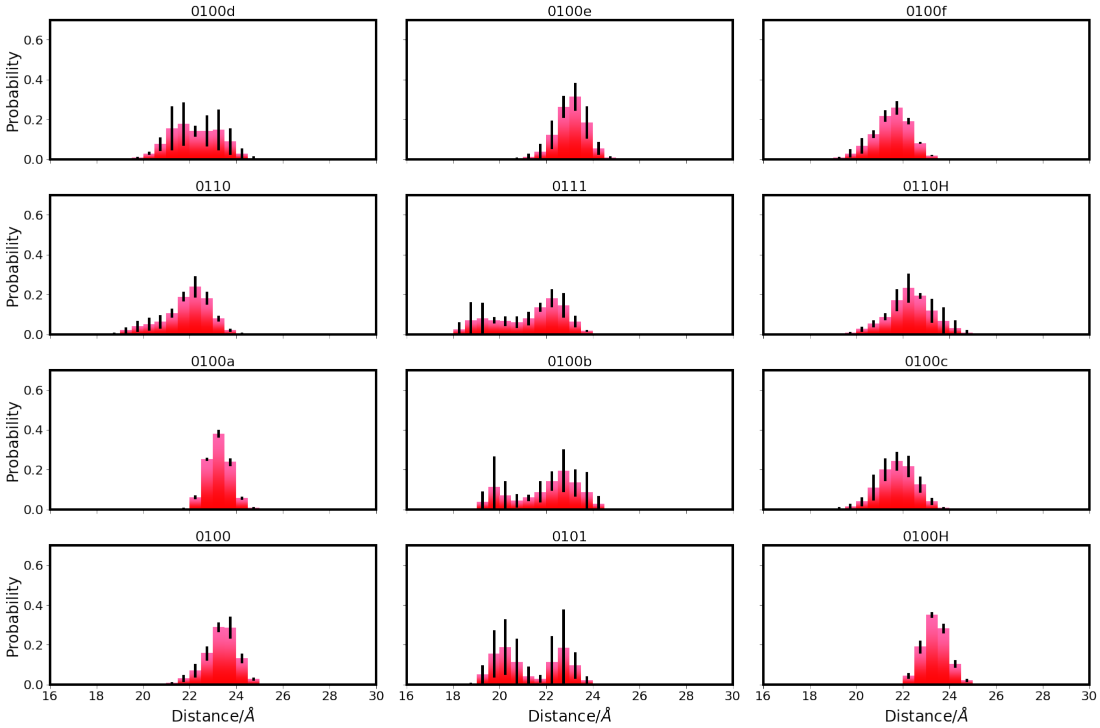

Figure S17: Probability distributions of distances between E101 and Y288.

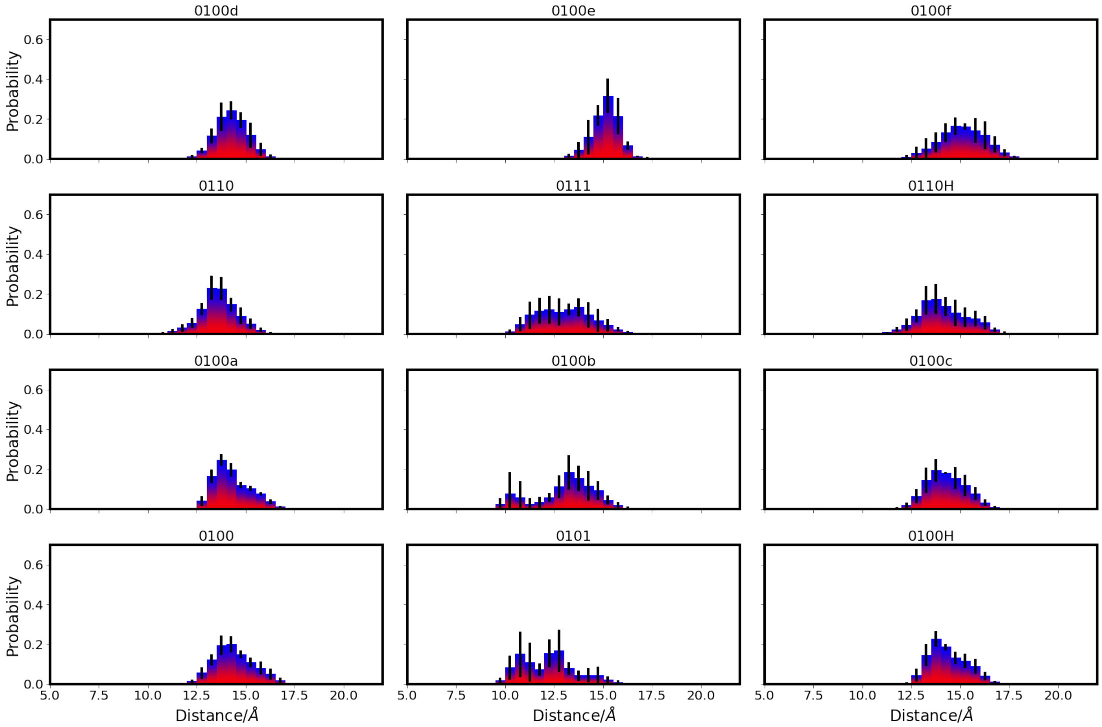

Figure S18: Probability distributions of distances between E101 and K362.

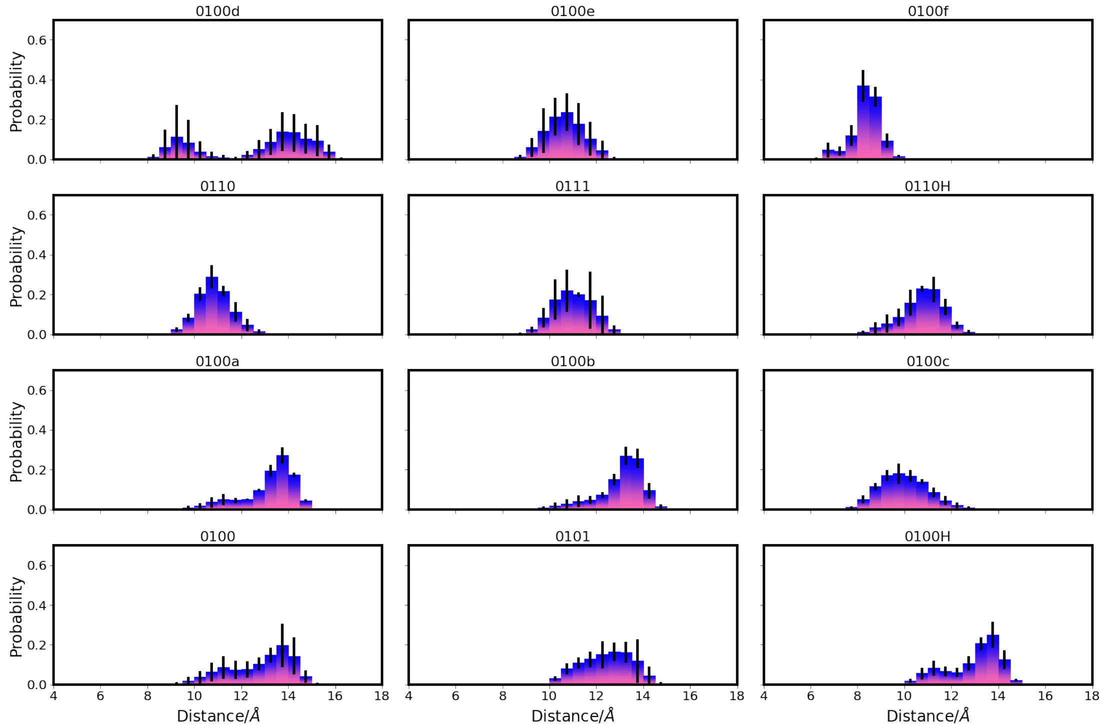

Figure S19: Probability distributions of distances between K362 and Y288.

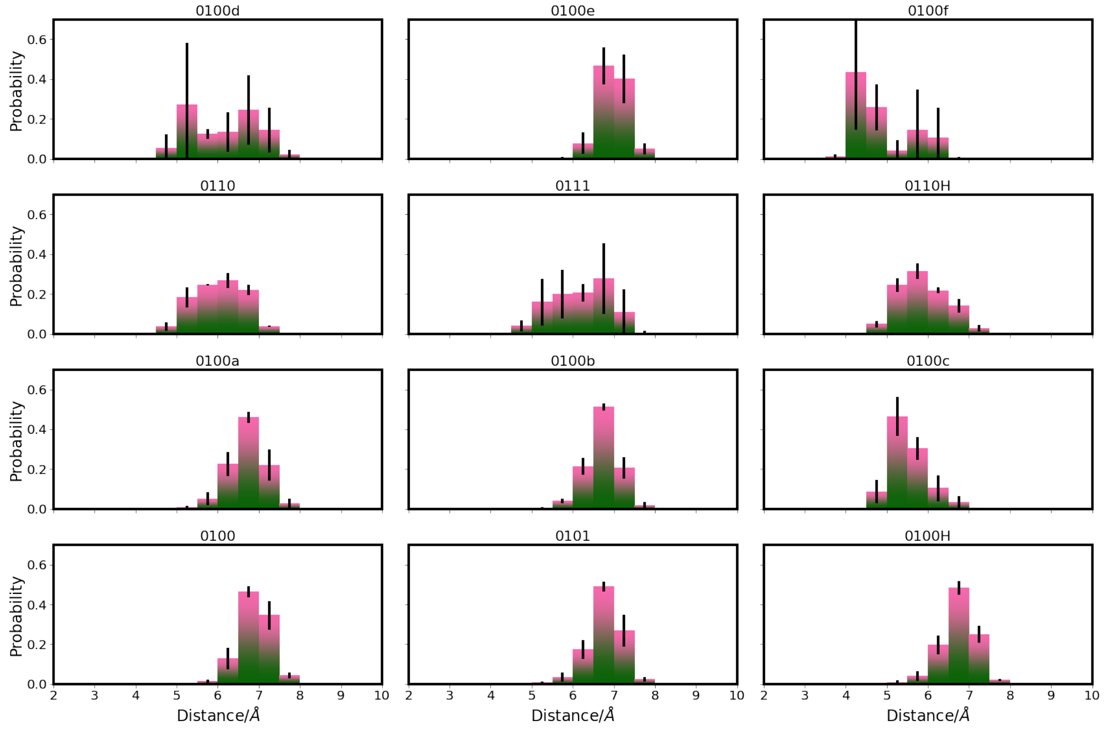

Figure S20: Probability distributions of distances between T359 and Y288.

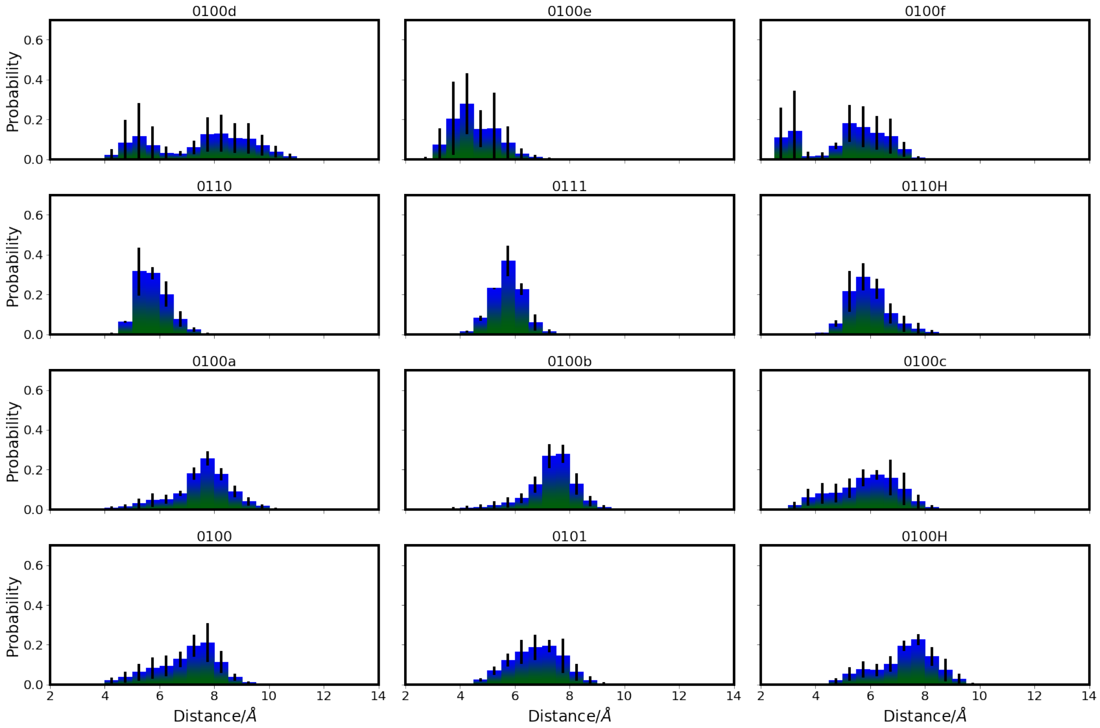

Figure S21: Probability distributions of distances between T359 and K362.

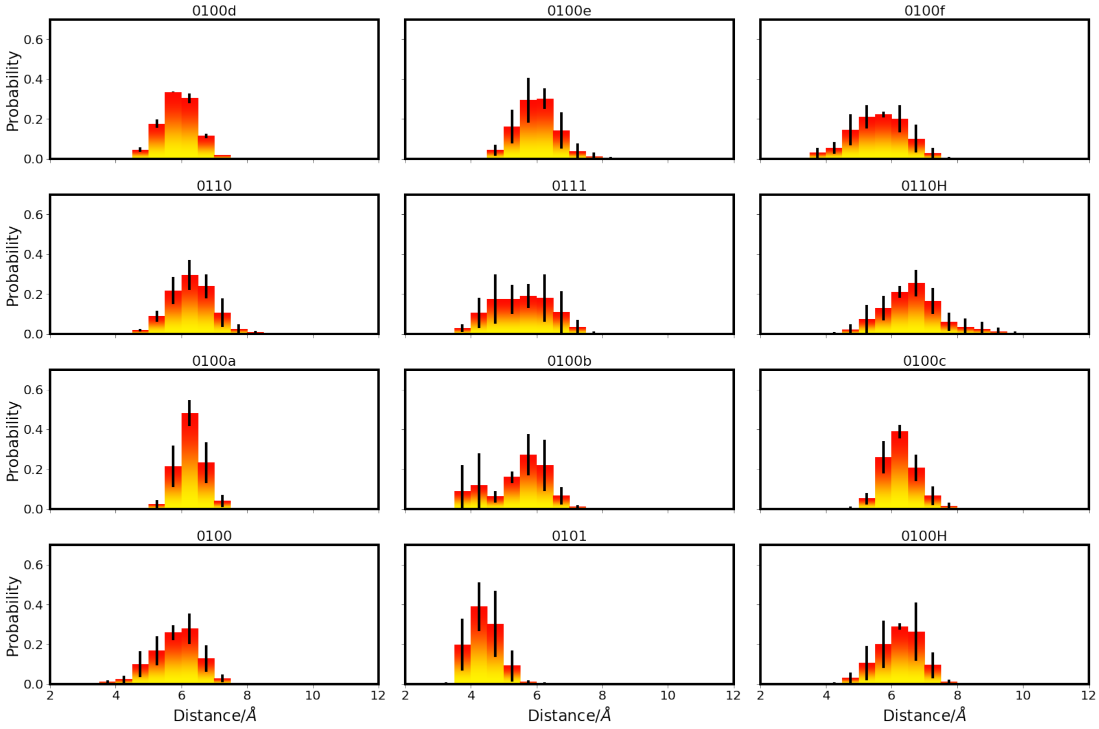

Figure S22: Probability distributions of distances between P315 and E101.

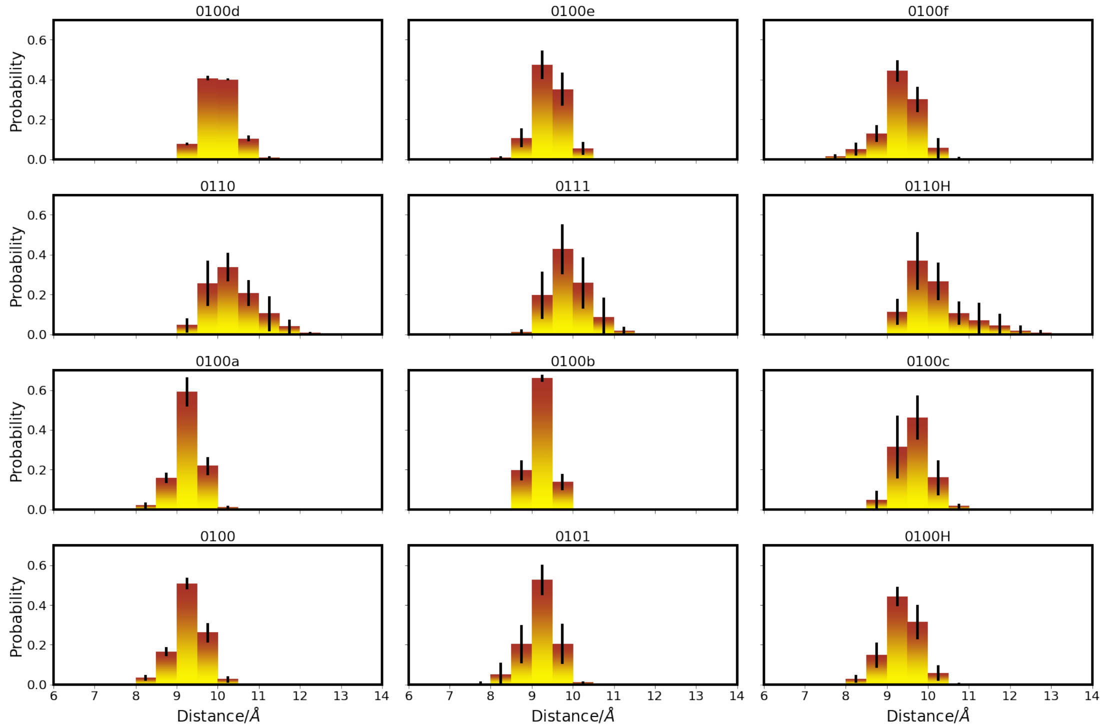

Figure S23: Probability distributions of distances between P315 and S365.

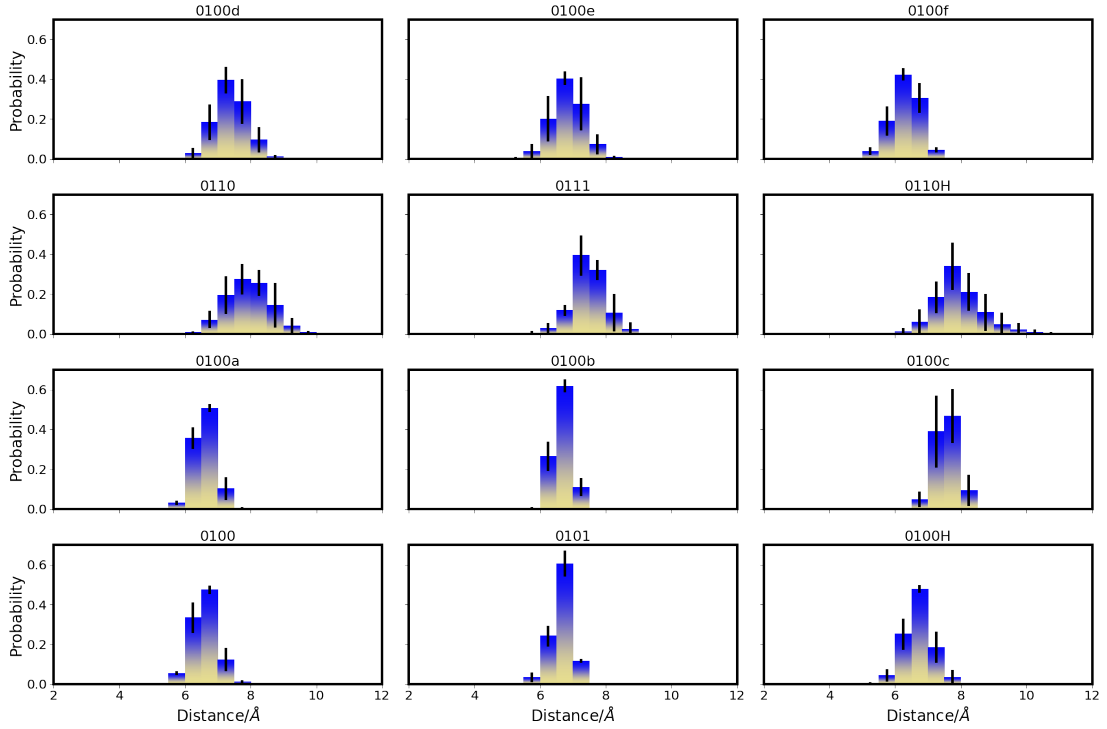

Figure S24: Probability distributions of distances between M316 and K362.

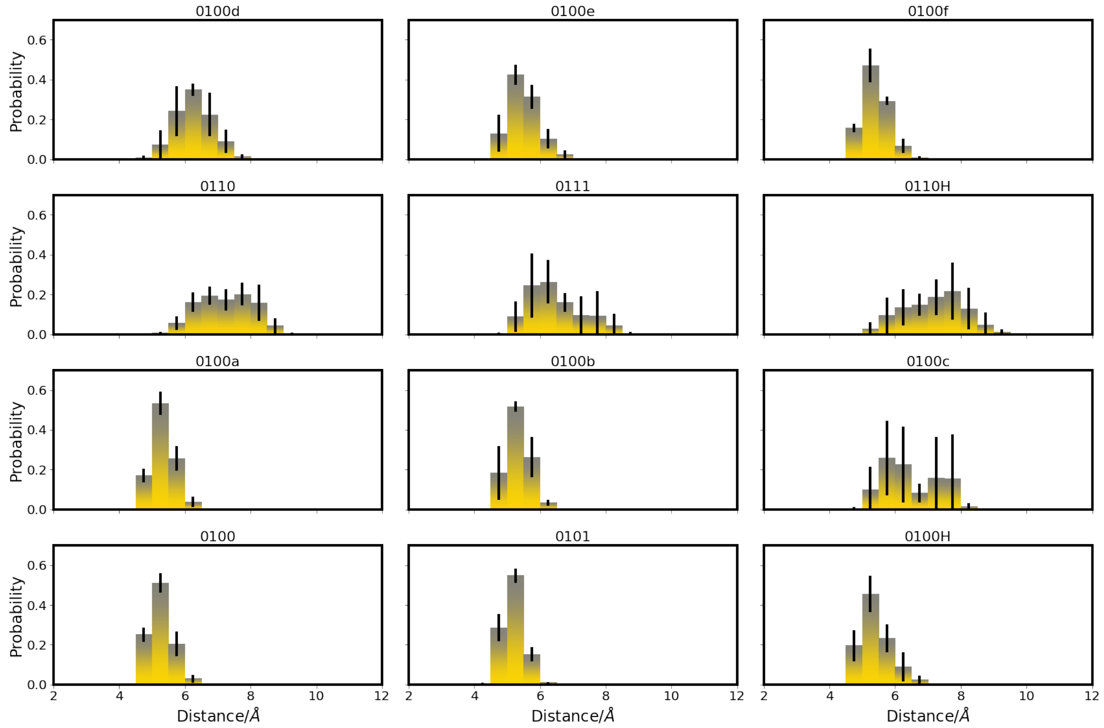

Figure S25: Probability distributions of distances between P358 and A319.
